# Supplementary material for: An Optimized Shotgun Strategy for the Rapid Generation of Comprehensive Human Proteomes
Source: Cell Syst. 2017 Jun 28;4(6):587–599.e4. doi: 10.1016/j.cels.2017.05.009 (PMC5493283; doi:10.1016/j.cels.2017.05.009)
Supplement: Document S1. Figures S1–S6 [file mmc1.pdf]

Cell Systems, Volume *4*

Supplemental Information

An Optimized Shotgun Strategy

for the Rapid Generation

of Comprehensive Human Proteomes

Dorte B. Bekker-Jensen, Christian D. Kelstrup, Tanveer S. Batth, Sara C. Larsen, Christa Haldrup, Jesper B. Bramsen, Karina D. Sørensen, Søren Høyer, Torben F. Ørntoft, Claus L. Andersen, Michael L. Nielsen, and Jesper V. Olsen

Figure S1

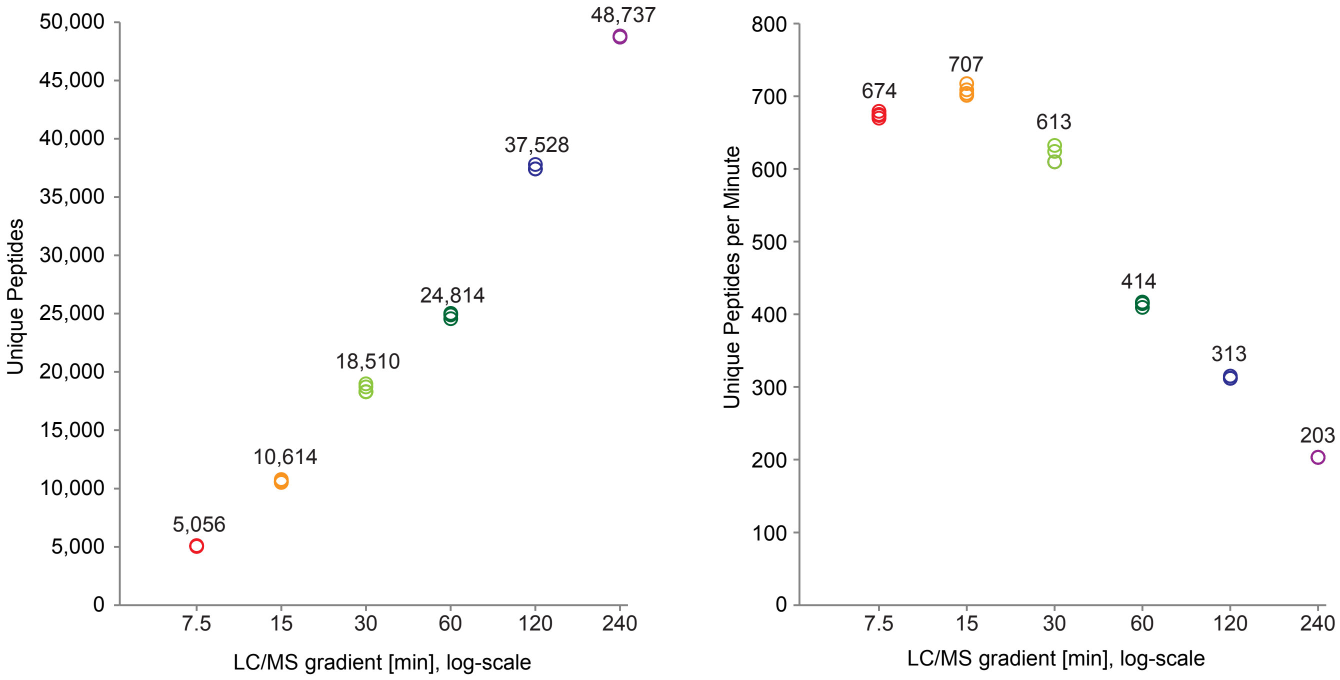

Figure S1: Relationship between LC/MS gradient length and peptide identification rates. Related to Figure 1. A fixed amount of 1ug Hela tryptic digest 'on column' was analyzed with variable gradient lengths. Optimal column and MS method was chosen for each time point. Left plot shows the total amount of unique peptides identified. The right plot shows the number of unique peptide identifications per minute. Above each time point the average of replica analysis is shown.

Figure S2

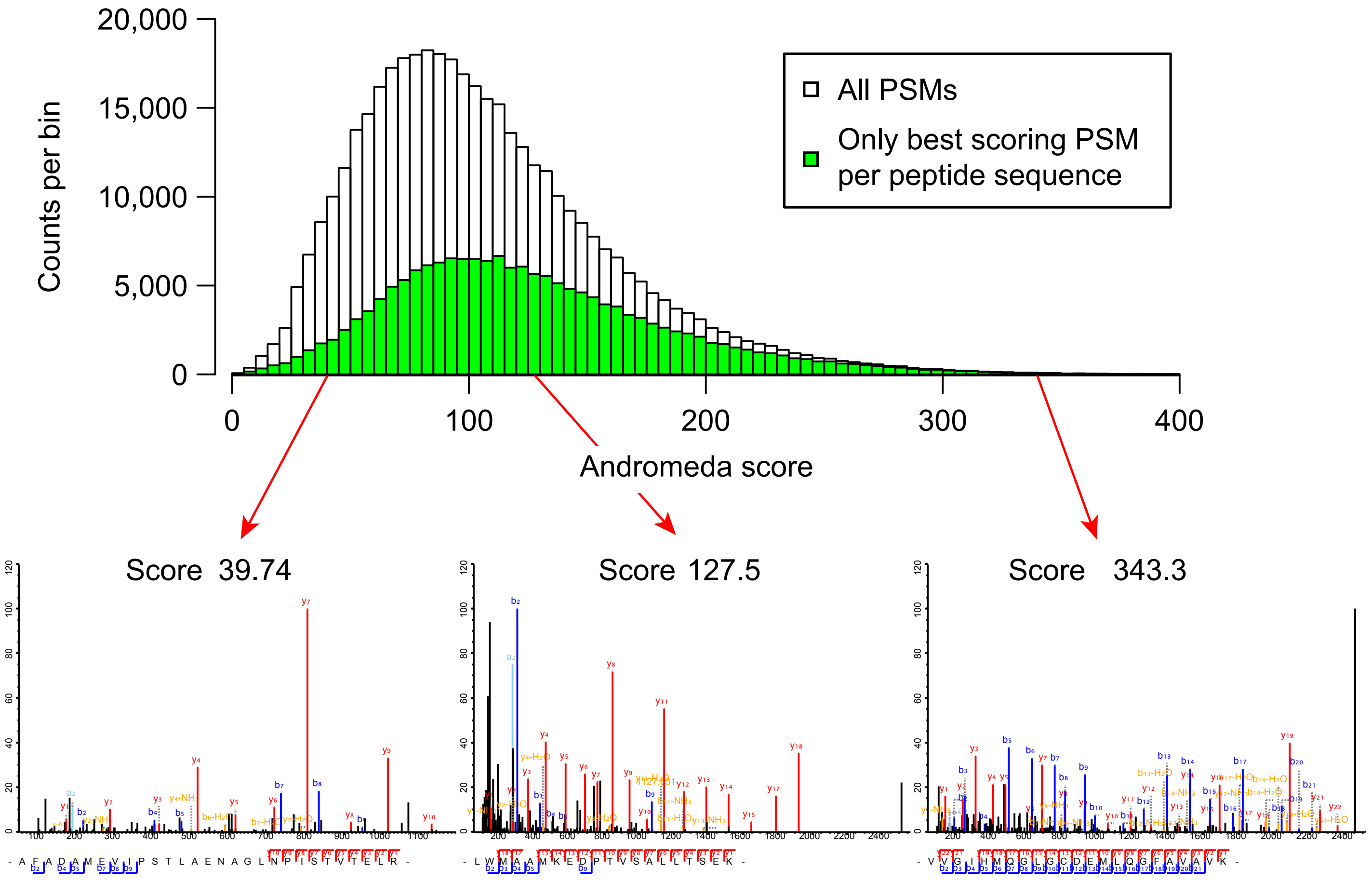

Figure S2: Score distributions for the HeLa experiment fractionated in 46 fractions. Related to Figure 1. The search engine (Andromeda) score distribution is shown for all PSMs and only the best scoring PSM per unique peptide sequence. Further, three annotated example spectra are shown for different scores.

Figure S3

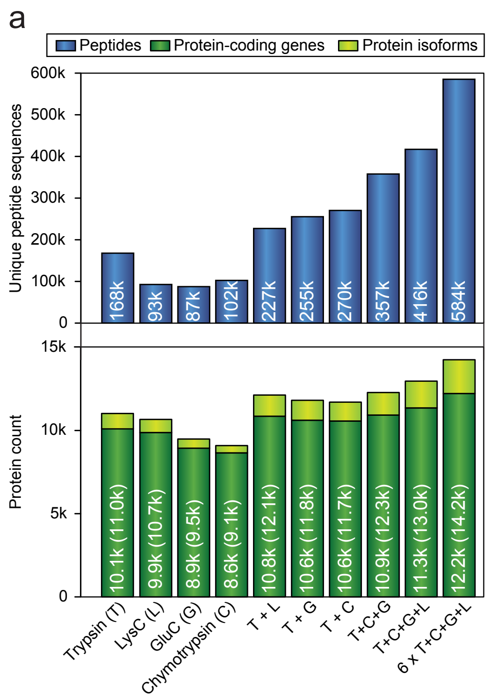

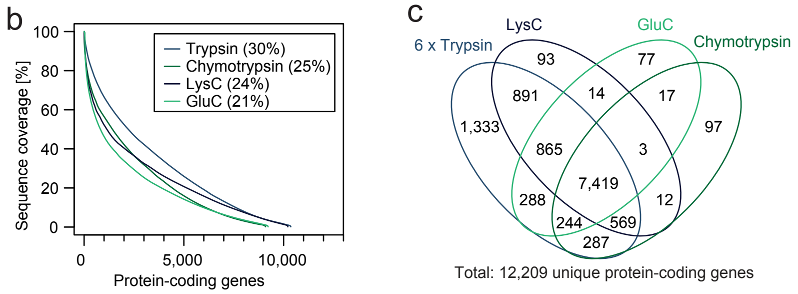

d

Protease	MS/MS	PSMs[a]	ID-rate	Unique Peptides[b]	Theoretical Peptides[c]
Trypsin	1,098,347	426,488	38.9%	168,569	1,866,929
Chymotrypsin	1,265,588	207,681	16.4%	102,311	2,646,893
GluC	1,252,604	249,340	19.9%	87,367	1,384,403
LysC	1,180,428	328,601	27.8%	92,546	1,151,077

[a]Non-redundant PSMs. [b]Unique peptide sequences. [c]Theoretical peptides obtained by in-silico digestion of 12,200 protein coding genes found in HeLa allowing up to 2 missed cleavages and a length above 6 amino acids.

Figure S3: Comparison of proteome coverage with different proteases. Related to Figure 3. a) Peptide and protein comparison using different enzymes. Single and two way combination with trypsin and technical replicates of digests are provided. b) Sequence coverage comparison of the different proteases. c) Venn diagram of protein coding genes between proteases. d) Comparisons of technical details associated with each protease. The identification rate is the proportion of peptide spectrum matches (PSMs) relative to the total number of MS/MS events.

Figure S4

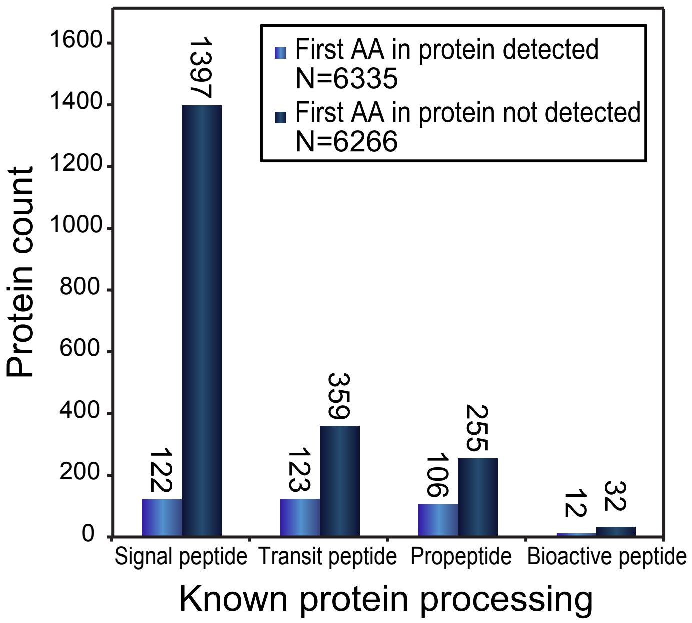

Figure S4: Analysis of protein N-terminus. Related to Figure 5. The two colours indicate whether an identified protein also had peptide sequence coverage of the first amino acid (AA) counted from the N-terminal of the protein. This analysis was extended to include different groups of known protein processing, obtained through annotation of the identified proteins from UniProt.

Figure S5

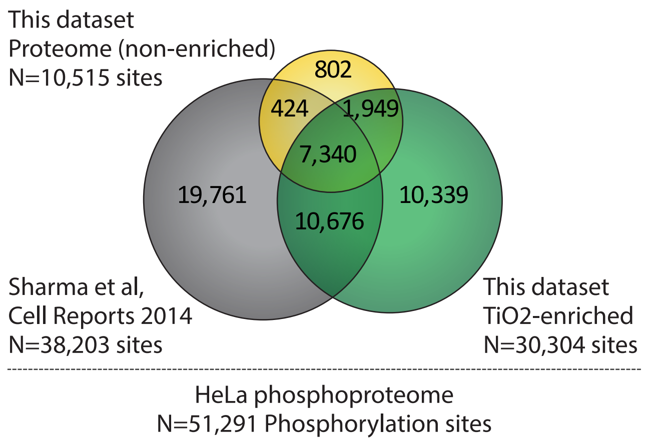

Figure S5: Phosphopeptides in HeLa. Related to Figure 5. Overlap of the phosphorylation sites foun with and without enrichment compared to the largest HeLa phosphoproteome published to date.

Figure S6

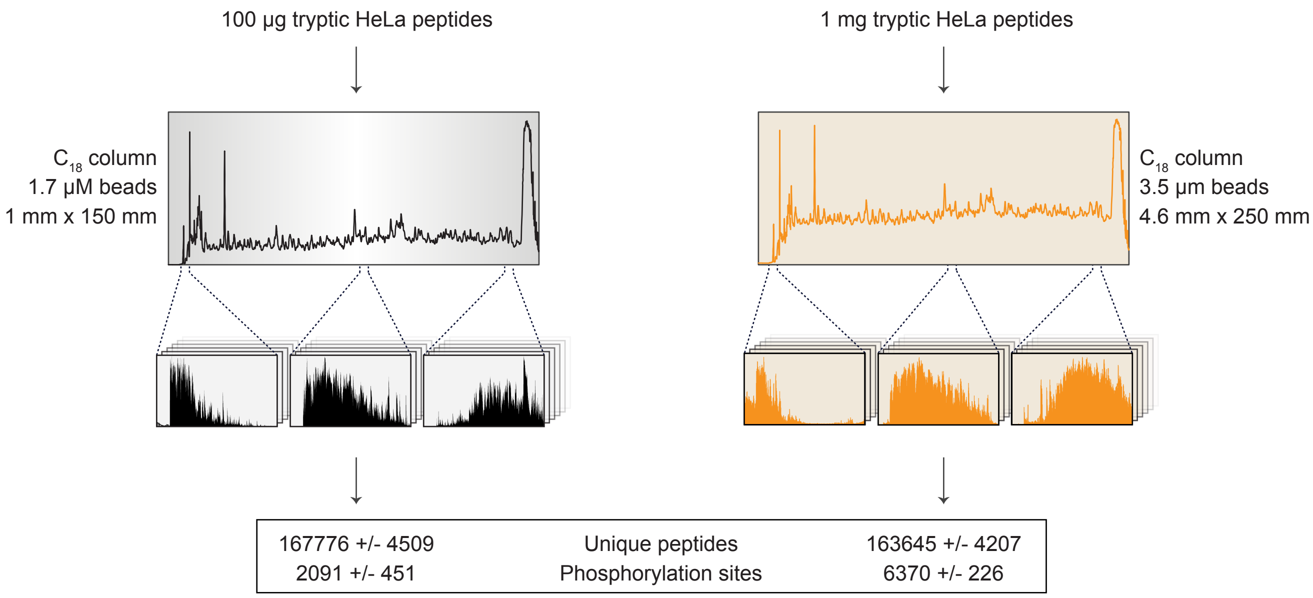

Figure S6: Downscaling of the peptide amount input. Related to Figure 7. Comparison of 100 µg tryptic HeLa peptides with 1 mg using the standard HpH 46 fractionation scheme.
