# Supplementary material for: An Optimized Shotgun Strategy for the Rapid Generation of Comprehensive Human Proteomes
Source: Cell Syst. 2017 Jun 28;4(6):587–599.e4. doi: 10.1016/j.cels.2017.05.009 (PMC5493283; doi:10.1016/j.cels.2017.05.009)
Supplement: Document S2. Article plus Supplemental Information [file mmc10.pdf]

Cell Systems

Article

An Optimized Shotgun Strategy for the Rapid Generation of Comprehensive Human Proteomes

Graphical Abstract

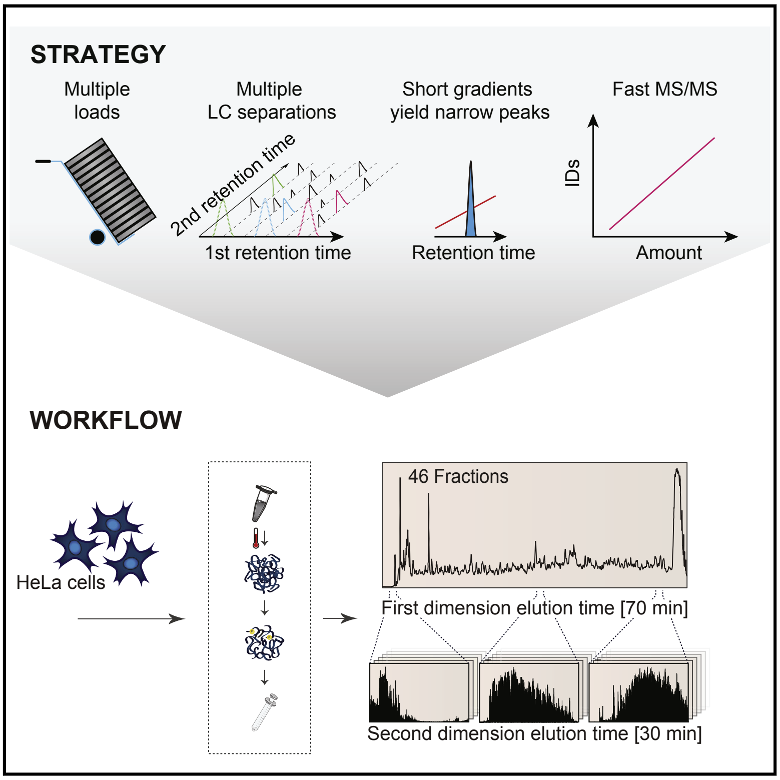

Highlights

- Multi-shot proteomics quantifies the protein levels of 12,200+ genes in HeLa cells
- This essentially complete HeLa proteome has coverage similar to next-gen RNA-seq
- Deep coverage of major PTMs is achieved without specific enrichment
- The approach is extendable to other human cell lines and patient samples

Authors

Dorte B. Bekker-Jensen, Christian D. Kelstrup, Tanveer S. Batth, ..., Claus L. Andersen, Michael L. Nielsen, Jesper V. Olsen

Correspondence

christian.kelstrup@cpr.ku.dk (C.D.K.), jesper.olsen@cpr.ku.dk (J.V.O.)

In Brief

Bekker-Jensen et al. show that proteomics can now provide an essentially complete HeLa proteome. They provide measurements acquired simultaneously for more than 12,200 protein-coding genes, 10,000 phosphorylation sites, and 7,000 N-acetylation sites. Their approach is fast, accessible, and requires modest amounts of starting material, making it easily extendable to other human cell lines and patient samples.

Bekker-Jensen et al., 2017, Cell Systems *4*, 587–599
June 28, 2017 © 2017 The Author(s). Published by Elsevier Inc.
http://dx.doi.org/10.1016/j.cels.2017.05.009

Cell Systems
Article

OPEN ACCESS
CellPress

An Optimized Shotgun Strategy for the Rapid Generation of Comprehensive Human Proteomes

Dorte B. Bekker-Jensen,[1,4] Christian D. Kelstrup,[1,4,*] Tanveer S. Batth,[1] Sara C. Larsen,[1] Christa Haldrup,[2] Jesper B. Bramsen,[2] Karina D. Sørensen,[2] Søren Høyer,[3] Torben F. Ørntoft,[2] Claus L. Andersen,[2] Michael L. Nielsen,[1] and Jesper V. Olsen[1,5,*]

[1]Proteomics Program, Faculty of Health and Medical Sciences, Novo Nordisk Foundation Center for Protein Research, University of Copenhagen, Blegdamsvej 3B, 2200 Copenhagen, Denmark

[2]Departments of Molecular Medicine and Clinical Medicine, Aarhus University Hospital, Aarhus University, Palle Juul-Jensens Boulevard 99, 8200 Aarhus, Denmark

[3]Institute of Pathology, Aarhus University Hospital, Palle Juul-Jensens Boulevard 99, 8200 Aarhus, Denmark

[4]These authors contributed equally

[5]Lead Contact

*Correspondence: christian.kelstrup@cpr.ku.dk (C.D.K.), jesper.olsen@cpr.ku.dk (J.V.O.)

http://dx.doi.org/10.1016/j.cels.2017.05.009

SUMMARY

This study investigates the challenge of comprehensively cataloging the complete human proteome from a single-cell type using mass spectrometry (MS)-based shotgun proteomics. We modify a classical two-dimensional high-resolution reversed-phase peptide fractionation scheme and optimize a protocol that provides sufficient peak capacity to saturate the sequencing speed of modern MS instruments. This strategy enables the deepest proteome of a human single-cell type to date, with the HeLa proteome sequenced to a depth of ~584,000 unique peptide sequences and ~14,200 protein isoforms (~12,200 protein-coding genes). This depth is comparable with next-generation RNA sequencing and enables the identification of post-translational modifications, including ~7,000 N-acetylation sites and ~10,000 phosphorylation sites, without the need for enrichment. We further demonstrate the general applicability and clinical potential of this proteomics strategy by comprehensively quantifying global proteome expression in several different human cancer cell lines and patient tissue samples.

INTRODUCTION

Comprehensive proteomics describes the mass spectrometric analysis of essentially all endogenously expressed proteins in a cell, tissue, or organism. This is the ultimate goal for proteomics studies, as it enables routine systems biology analyses and biomarker discoveries (Eriksson and Fenyo, 2007). Tremendous efforts by the proteomics community have already enabled essentially complete proteome analysis of simpler organisms, such as Baker's yeast, with the first comprehensive analysis of the yeast proteome achieved 9 years ago (de Godoy et al., 2008). While it required a week of total mass spectrometry (MS) instrument time to obtain this original data, the complete yeast proteome can now be analyzed a hundred times faster, in 1.8 hr (Hebert et al., 2014).

Efforts to map the complete human proteome have likewise mainly been attempted by brute force analyses, where very large-scale experiments have been carried out. This was, for example, the case in the two recent initial drafts of the human proteome, where thousands of MS analysis runs were combined (Kim et al., 2014; Wilhelm et al., 2014). However, these datasets also sparked a debate in the proteomics community about how to control false discovery rates (FDRs) on the level of individual proteins; that is estimating and minimizing error rates by correcting for multi-hypothesis testing in large-scale datasets. Recent statistical developments and re-analysis of the data has since lowered the original claims of proteome coverage (Ezkurdia et al., 2014; Savitski et al., 2015; The et al., 2016). While these reanalyzed proteome drafts demonstrate that comprehensive coverage of the human proteome is possible even when FDR is controlled for, they achieved these high protein numbers by combining analyses of many different cell types, i.e., the proteome coverage of a single-cell type was not extraordinary compared with other large-scale studies (Iwasaki and Ishihama, 2014).

Deep proteome analysis of single human cell types has been done by so-called "single"-shot analysis where a single-dimension liquid chromatography (LC) is coupled to MS (LC-MS) (Geiger et al., 2012). While "single"-shot analysis is preferable due to its simplicity and robustness, it will not achieve the same depth compared with classic multidimensional fractionation strategies where multiple fractionation schemes are used in series such as LC/LC/MS and higher. This is due to the fact that fractionation of peptides and proteins is a simple way to increase peak capacity of the LC separation (Wolters et al., 2001). In particular, offline peptide fractionation at high pH (HpH) prior to low pH online in an LC/LC-MS setup has shown great promise in recent years (Mertins et al., 2016; Wang et al., 2011). Another emerging trend in deep proteome analysis has been to make use of ever longer online peptide separation gradients to boost identification numbers, but to benefit from this requires extreme

CrossMark

This is an open access article under the CC BY license (http://creativecommons.org/licenses/by/4.0/).

OPEN ACCESS
CellPress

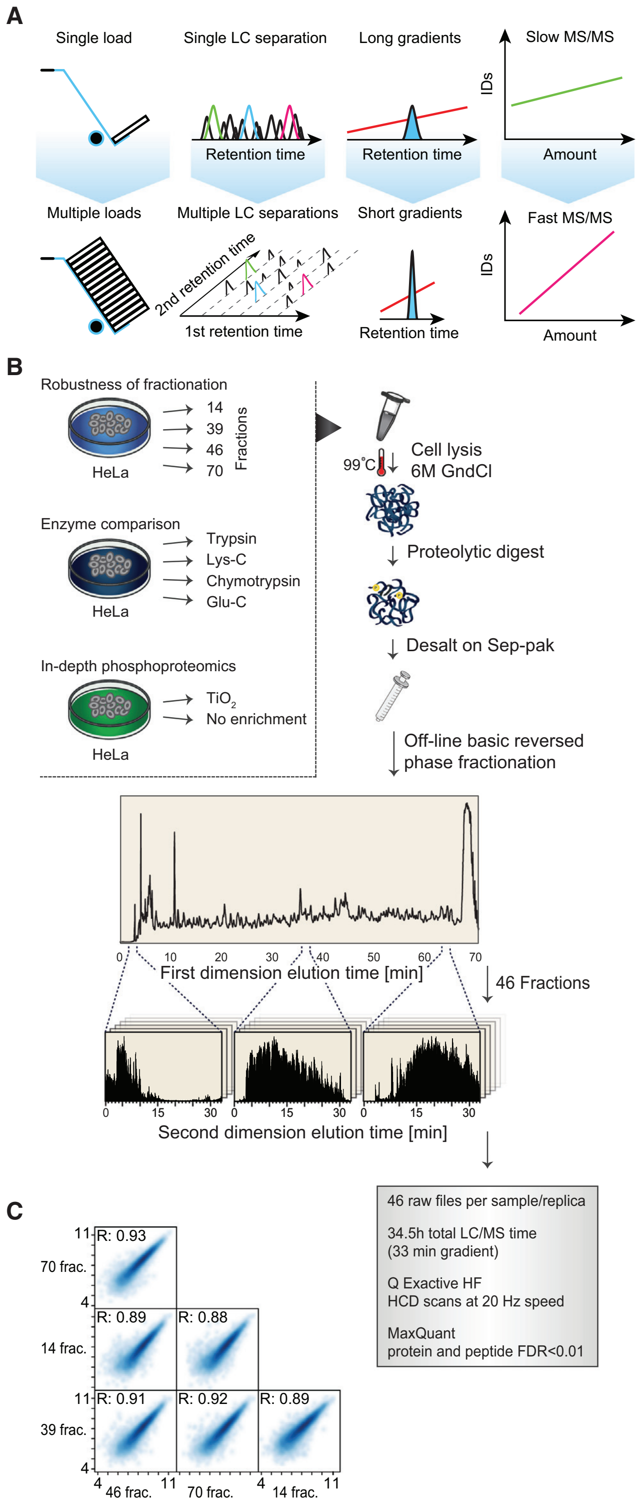

Figure 1. Workflow Overview

(A) Conceptual strategy for improving the limit of detection through multiple sample injections, increasing the peak capacity through multiple LC separations, maximizing ion flux and instrument time using short LC-MS gradients, while keeping the MS in the fastest scanning mode.

(B) Experimental workflow of all HeLa experiments.

(C) Quantitative reproducibility of the method.

chromatographic performance using very long analytical columns (Iwasaki et al., 2012; Nagaraj et al., 2012). We have previously argued against longer gradients as this increases the time window within which a peptide elutes and thereby naturally dilutes its apex peak intensity or ion flux, shifting the MS analysis optimum to slower scanning MS methods (Kelstrup et al., 2012).

Here, we present a readily approachable, optimized method for generating deep proteomes. This method takes full advantage of the high resolution that offline HpH fractionation provides and combines it with short gradients and a fast 20 Hz scanning tandem MS (MS/MS) method for subsequent proteome analysis. This combination solves the problems of lower sensitivity usually associated with fast scanning MS/MS methods, and we demonstrate that the concept of running many fractions on short gradients is the sweet-spot in terms of best compromise between instrument time used and sequencing depth obtained. To benchmark this workflow we analyzed the human proteome of the HeLa cervix carcinoma cell line, which is the most commonly used model for studying human cell biology (Masters, 2002). HeLa cells are also the most widely used human cell line applied in proteomics studies (von Stechow et al., 2015), making it the ideal model system for a reference proteome. Collectively we identify more than 14,000 unique protein groups covering 12,200 protein-coding genes and demonstrate that the HeLa proteome can be comprehensively analyzed to a depth similar to that of next-generation RNA-seq technology. This method is generally applicable, in that it accurately quantifies the proteomes of other human cell lines as well as patient tissue biopsy samples with similar proteome depth. Finally, we demonstrate that the massive peptide sequencing simultaneously yields deep coverage of the major post-translational modifications (PTMs) without specific enrichment.

RESULTS

A multi-shot proteomics strategy is known to increase the dynamic range and coverage compared with single-shot experiments in human proteome investigations. We hypothesized that a strategy based on high sample amounts and offline peptide pre-fractionation collecting high numbers of fractions in combination with short LC-MS/MS gradients and high peptide sequencing speed would overcome many of the inherent dynamic range issues in proteomics without compromising the overall analysis time required. We reasoned that optimization and maximization of each of the four interconnected technical parameters were needed (Figure 1A). First, we wanted to improve the detection limit by increasing total peptide amounts analyzed. Since the maximal loading capacity of our online nano-scale LC-MS system, without significant loss in chromatographic performance or peptide identifications (Kelstrup et al., 2014), is around 1 μg on the column, the use of multiple injections is a way to indirectly increase the loading capacity. Second, to increase peak capacity and effectively separate milligrams of complex peptide mixtures into multiple fractions we utilized high-capacity offline HpH reversed-phase LC with high resolving power generating numerous (39–70) fractions containing large peptide amounts and with minimum overlap between individual fractions. Third, to decrease peak widths and increase MS signal intensity of analyzed peptides and simultaneously decrease

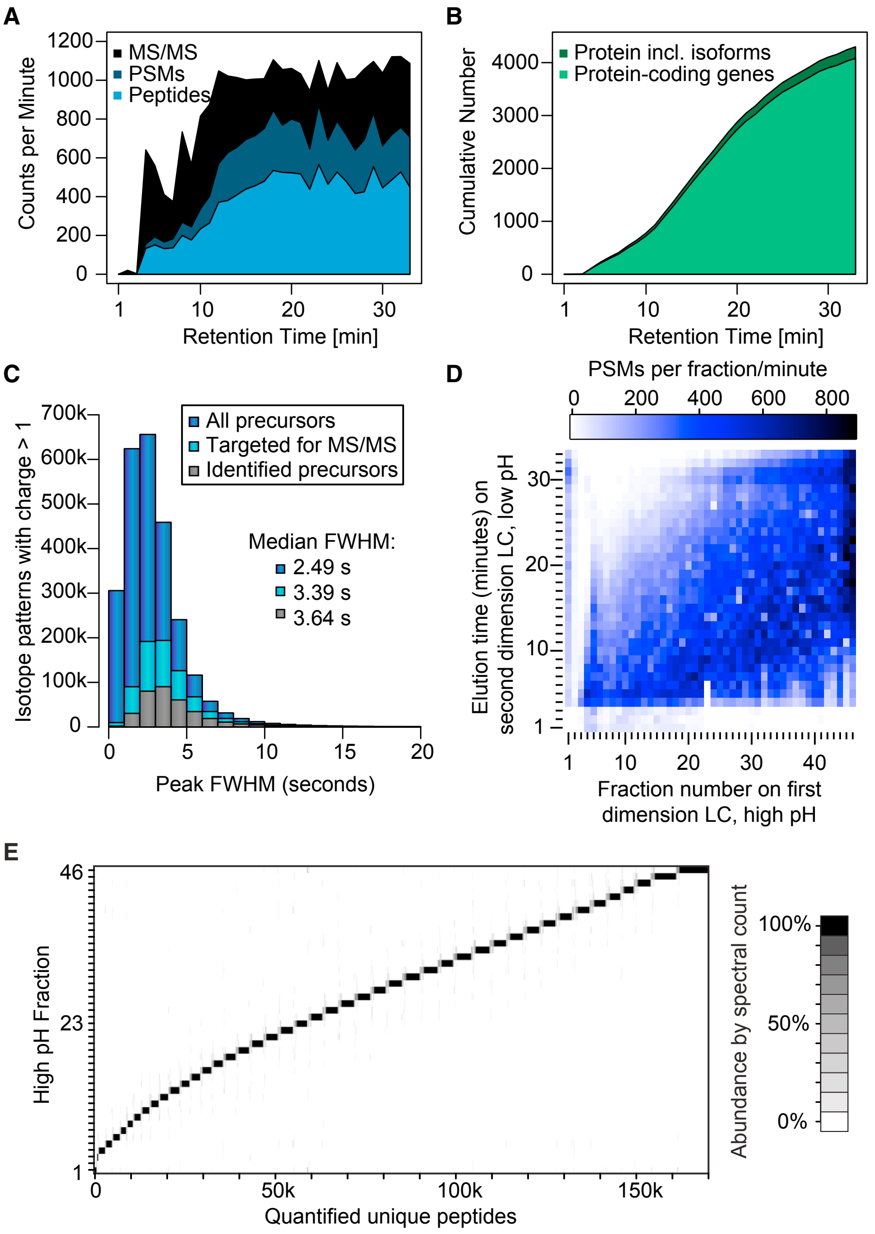

Figure 2. Workflow Performance Characteristics

(A) Visualization of peptide sequencing speed analyzing HpH fraction 46.

(B) Cumulative number of proteins and protein-coding genes through HpH fraction 46.

(C) Histogram illustrating LC peak width distributions for all multi-charged isotope patterns, targeted precursors, and identified peptides.

(D) Heatmap representing the orthogonality of LC separations through binning of both dimensions by minutes.

(E) Visualization of peptide overlap between HpH fractions.

overall MS analysis time, we optimized a short 30 min gradient for online LC-MS, and minimized the loading, washing, column equilibration, and other overheads between injections to 15 min. The 30 min gradient provided the best compromise between maximizing the number of unique peptides identified, while maintaining high identification rates when benchmarked against longer and shorter gradients (Figure S1). Fourth, to cope with the fast chromatographic separations we made use of the fastest scanning higher-energy collisional dissociation (HCD) (Olsen et al., 2007) MS/MS with 20 Hz orbitrap acquisition rates. This method leveraged the increased MS signal intensities, thereby achieving overall peptide identification rates of approximately 40% with high identification scores (Figure S2).

We initially optimized peptide fractionation and MS analysis workflow with total cell lysates derived from the commonly used HeLa cell line, testing different fractionation schemes and proteases (Figure 1B). In brief, adherent HeLa cells were efficiently lysed by boiling directly in a GndCl buffer and extracted proteins digested overnight. Aliquots of 1 mg of the resulting peptides were fractionated by offline HpH reversed-phase chromatography (Batth et al., 2014) without fraction concatenation, and each fraction was analyzed by online LC-MS/MS using a Q-Exactive HF orbitrap instrument in the fastest HCD scanning mode (Kelstrup et al., 2014) (Figure 1B). Analyzing 1 μg of HeLa peptides on the column from each of 46 HpH fractions with 30 min LC-MS/MS gradients (45 min run-to-run), using a total of 34.5 hr resulted in identification of 166,620 unique peptide sequences covering 11,292 protein groups or 10,284 protein-coding genes. No matter how the sample was fractionated, reproducibility in terms of measured absolute protein abundances between experiments was high with Pearson correlation coefficients of around 0.9 (Figure 1C). To determine the inter-sample variability, we compared the two biological HeLa replicates, 46 and 70 fractions, respectively, and found roughly half of the proteins to have coefficients of variation of less than 20%.

A key aspect to achieve this deep proteome coverage was the high peptide loads together with short gradients and a very fast scanning speed of the instruments, with more than 1,000 MS/MS per minute, of which more than 40% on average could be identified (Figure 2A). If the utmost speed in analysis is required, it is possible to identify more than 4,000 protein groups in a single fraction (Figure 2B). The fast gradient together with high-resolution online chromatography enabled a median peak full width half maximum of 3.64 s of identified precursors. Assuming a roughly Gaussian peak shape and a classic definition of full peak width equal to 4 SDs, the median peak full width is on average 6.2 s and the resulting peak capacity for each fraction can be estimated around 290 (Figure 2C). This is not

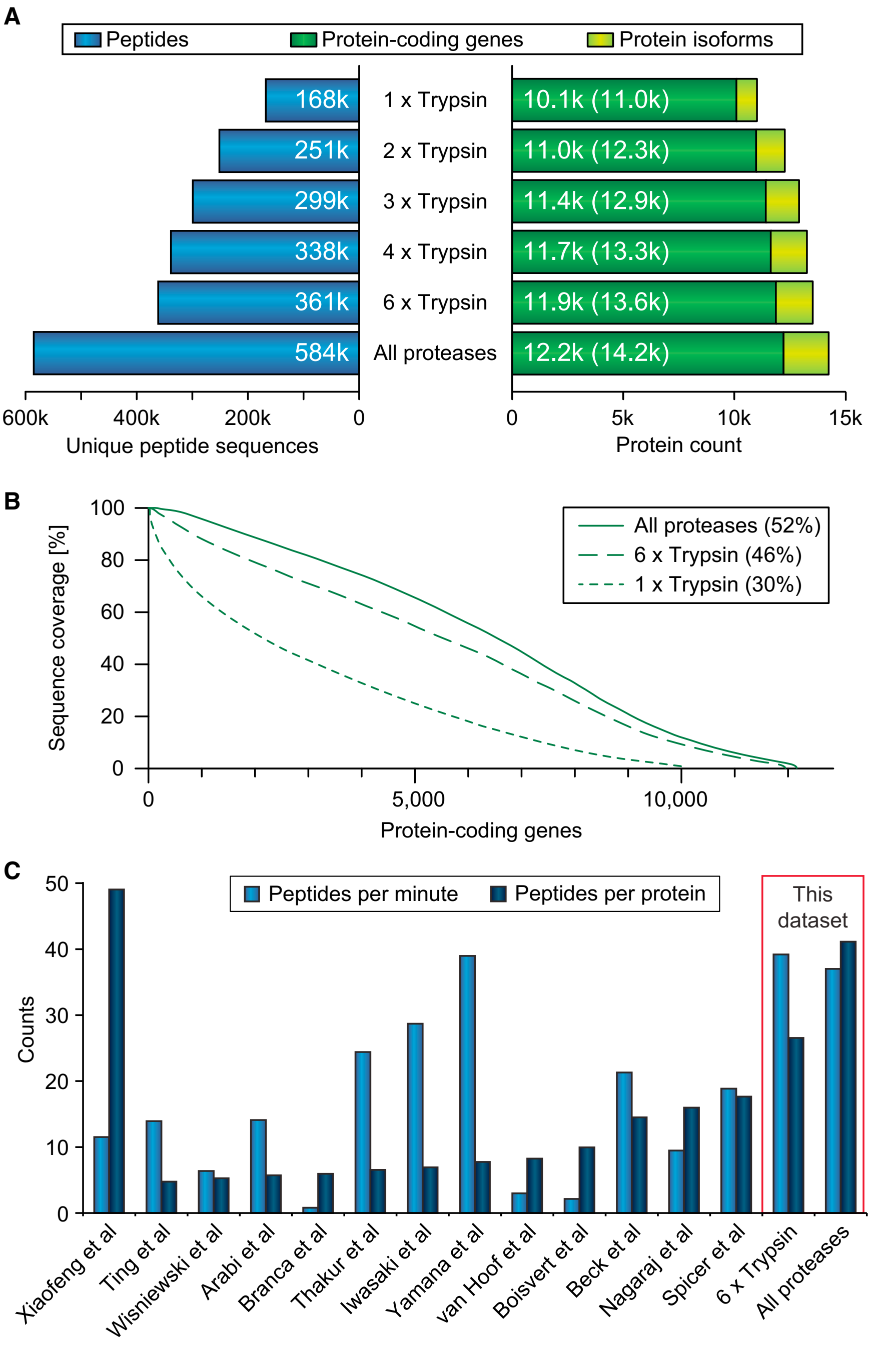

Figure 3. Comprehensive Analysis of the HeLa Proteome

(A) Identifications based on replica digests and alternative proteases for peptides, protein-coding genes, and proteins, including isoforms.

(B) Comparison of sequence coverage achieved.

(C) Benchmarking this HeLa dataset against other published datasets of deep single-cell proteomes. Bar chart showing comparisons of unique peptide sequences identified per minute of analysis time and peptides per protein.

extraordinarily impressive, but when 46 fractions are analyzed together this translates into a very high peak capacity estimation of 13,300. The lack of full orthogonality between separations in the two LC dimensions lowers the actual peak capacity value (Figure 2D), but it is still one order of magnitude higher than the best one-dimensional LC separations that may approach a peak capacity of 1,000 on long columns/gradients (Shishkova et al., 2016). The offline HpH fractionation was performed with high resolution, as 82% of peptide spectrum matches and 75% of peptide sequences were unique to a single HpH fraction, and a further 17% of peptide sequences were only found in two fractions as indicated by the gray scale in the graphical representation (Figure 2E). When increasing the number of fractions from 46 to 70, the peptide sequences unique to one fraction dropped from 75% to 61%, and the corresponding peptide and protein identifications are therefore not vastly higher, but total analysis time needed increased by 50% (Table S1).

Utilizing alternative proteases to trypsin has previously been demonstrated as a means to reveal an undetected part of the proteome and increase proteome coverage (Giansanti et al., 2015, 2016; Guo et al., 2014; Low et al., 2013; Swaney et al., 2010). Therefore, we tested the benefits of using Glu-C, Lys-C, and chymotrypsin as alternative proteases to trypsin using the multi-shot proteomics workflow. For a single sample, trypsin identified the highest number of both peptides and proteins (Figure S3). This is not surprising as trypsin is historically the preferred protease used in most shotgun proteomics studies due to its properties of generating peptides with predictable cleavage patterns (Olsen et al., 2004) that fragment very well in MS. When combining the analyses of all six tryptic digests, we identify 361,000 unique tryptic peptide sequences mapping to 11,900 unique protein-coding genes and 13,600 unique proteins. Adding the additional 223,000 unique peptides from the three other proteases results in a total of 584,000 unique peptide sequences from HeLa cells (Table S2), which is more than those presented in the recent organ-wide drafts of the human proteome (Kim et al., 2014; Wilhelm et al., 2014) (Table S1). The unique peptides from the additional proteases increased the total number of covered protein-coding genes by 300 to a total of 12,200 and they provided increased coverage of protein isoforms by twice as much to a total of 14,200 (Figures 3A and S3). Another way to evaluate proteome coverage is by calculating the average amino acid coverage per protein achieved. Adding the 223,000 unique peptide sequences from the alternative protease datasets increases

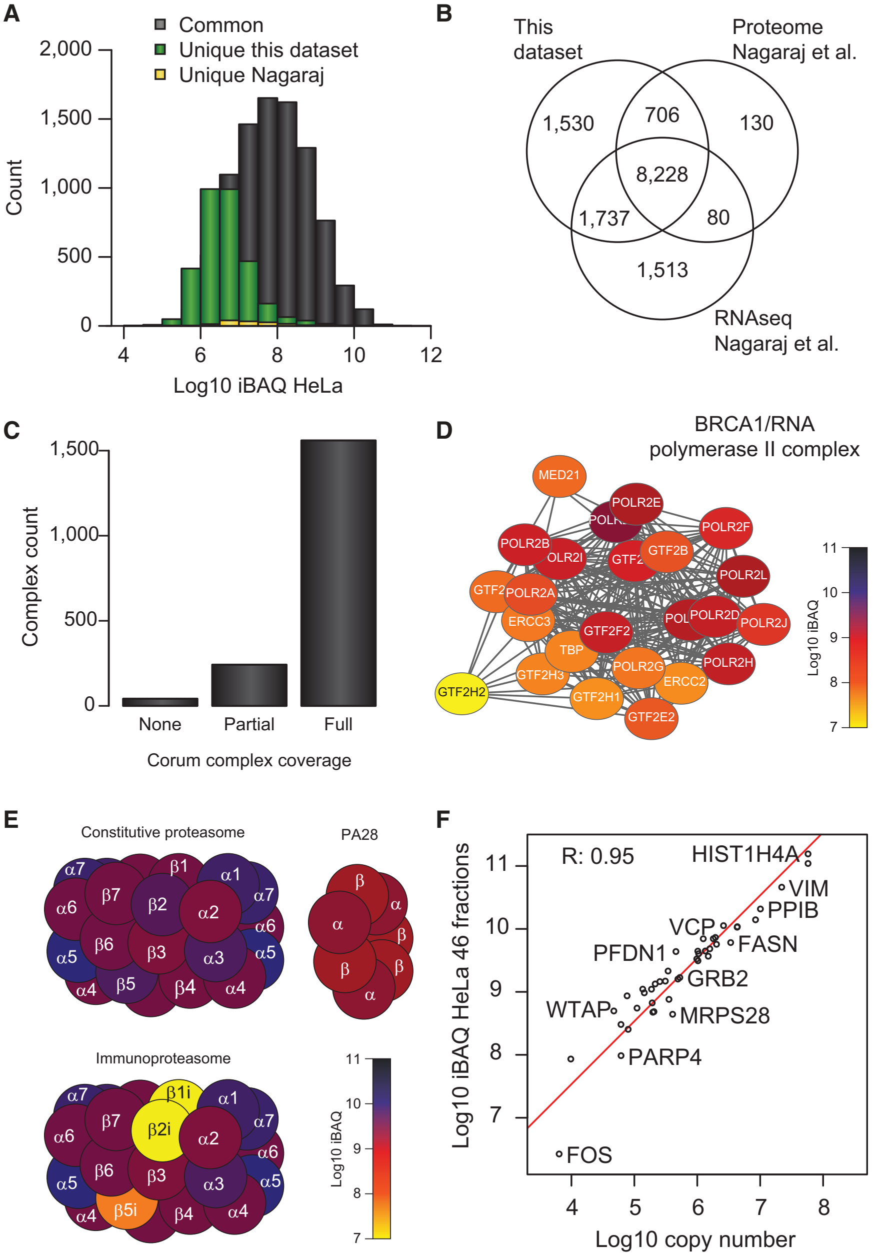

Figure 4. Functional Analysis of the HeLa Proteome

(A) Comparison of protein-coding gene abundances in HeLa identified in this dataset with the largest existing HeLa proteome published so far.

(B) Comparison of protein-coding genes identified in HeLa in this dataset with previously published proteome and next-generation RNA-seq data of HeLa cells.

(C) CORUM protein complex coverage of the identified proteins in this dataset.

(D) Abundance of BRCA1/RNA polymerase II complex members in HeLa cells visualized according to their individual protein intensities.

(E) Abundance of proteasomal proteins in HeLa visualized according to their individual protein intensities.

(F) Scatterplot of HeLa protein copy-number estimation from this dataset with previously published copy numbers.

the average sequence coverage to 52% (Figure 3B). A simple amino acid count of the 584,000 unique peptides relative to the count of sequences from the identified protein-coding genes results in a ratio of 1.44, also highlighting that a significant overlap is observed among the peptide sequences.

Previously published studies (Arabi et al., 2012; Beck et al., 2011; Boisvert et al., 2012; Branca et al., 2014; Guo et al., 2014; Iwasaki et al., 2012; Nagaraj et al., 2011; Spicer et al., 2016; Thakur et al., 2011; Ting et al., 2011; Van Hoof et al., 2009; Wisniewski et al., 2009; Yamana et al., 2013) have also provided deep proteome coverage of single human cell types with high peptide coverage. However, this has typically been done at the expense of pro-longed acquisition time, whereas studies focusing on high protein coverage using shorter MS analysis time generally results in low overall peptide coverage. Conversely, our study demonstrates that this analytical strategy makes it possible to achieve high protein coverage with short MS analysis time (Figure 3C).

The combined deep HeLa proteome presented here expands the previous record by a third, as more than 3,000 low abundant protein-coding genes are now identified of which many are receptors and transcription factors (Figure 4A and Table S3). Furthermore, our HeLa dataset of 12,200 protein-coding genes have comparable coverage with next-generation RNA-seq data of the HeLa cells (Figure 4B) (Nagaraj et al., 2011). RNA-seq provided evidence for expression of close to 90% of the HeLa proteins detected by MS and vice versa. As expected, the unique subsets of both datasets are biased toward low abundance.

Although these analyses suggest that our coverage of the HeLa proteome is quite complete, an alternative way to assess the completeness of a proteome is to quantify the coverage of known macromolecular protein complexes. Most macromolecular complexes were completely covered in our HeLa proteome, and typically more than 90% of the members of low-abundance cellular machineries were quantified, as estimated by the detection of known protein complexes described in the CORUM database (Ruepp et al., 2008) (Figure 4C and Table S4). The complexes listed within CORUM that are not present in HeLa cells, which were originally taken from cancerous cervix, are largely cell-type-specific complexes encompassing neuronal and immune specific proteins. Combining the protein abundance estimates for proteins with information about the macromolecular complex they belong to can be quite informative as this indirectly reveals the stoichiometry of individual

complex components. This is exemplified by the BRCA1/RNA polymerase II complex (Figure 4D) and the broad abundance distribution of proteins belonging to the different proteasome types (Figure 4E). For example, the three specific subunits of the immunoproteasome are orders of magnitude lower in expression compared with their counterparts in the constitutive proteasome (Figure 4E). Consequently, the immunoproteasome must be at least a 100-fold less abundant than the constitutive proteasome in HeLa cells. Moreover, there is a significant abundance difference between the individual immunoproteasomal subunits as PSBM8 (b5i) is 10-fold more abundant than the two other subunits, PSBM9 (b1i) and PSBM10 (b2i). This supports the presence of multiple intermediary immunoproteasomal units as previously suggested (Guillaume et al., 2010). Based on these comparative analyses of mRNA data and protein complexes, we conclude that the expressed HeLa proteome analyzed here is essentially complete.

Such quantitative analysis enables absolute quantitation of protein copy numbers. The intensity-based absolute quantitation (iBAQ) of proteins analyzed by shotgun proteomics data has been shown to provide a reasonably accurate estimate of protein copy numbers (Schwanhausser et al., 2011). The iBAQ values are calculated by summing all peptide intensities for a protein and dividing this by the number of theoretical tryptic peptides between 6 and 30 amino acids in length to correct for protein size. Moreover, precise copy numbers for a small set of proteins in HeLa cells spanning the entire expression range have been established (Hanke et al., 2008; Wisniewski et al., 2014; Zeiler et al., 2012). Comparing the protein iBAQ values calculated for our complete HeLa dataset with the previously established HeLa protein copy numbers in log-space, we find a strong linear correlation with Pearson correlation coefficient of 0.95 (Figure 4F). Accordingly, from this we can determine the copy number of all 12,000 quantified proteins in HeLa cells simply by subtracting a $\log_{10}$ value of roughly 3.3 from our $\log_{10}$-transformed iBAQ values.

Deep PTM Analysis without Enrichment

This comprehensive proteome also facilitates observation of PTMs, including specific proteolytic cleavage, phosphorylation, and N-acetylation. For example, the N terminus of proteins is usually the most accessible part and this is often post-translationally processed by proteolytic cleavage of a signal peptide or modified by acetylation. Determining the sequence and nature of protein N termini therefore provides important functional annotation of proteins. We identified the peptide covering the N termini of more than half of the proteins and the absence of detection of N termini peptides from approximately 2,000 proteins can readily be explained by known proteolytic processing events such as removal of signal peptides and transit peptides (Figure S4).

Although global analysis of any PTM by MS/MS usually requires specific enrichment of the PTM-bearing peptides from total cell digests, we speculated that large-scale identification of PTMs without specific enrichment should be possible. To test this hypothesis, we searched our HeLa dataset for the major intra-cellular PTMs, including site-specific phosphorylation, acetylation, and methylation. From this analysis we identified 18,237 unique PTM sites (Table S5), including more than 10,000 phosphorylation sites and more than 5,000 N-acetylation sites. For protein N-acetylation, the coverage of sites is at the same level as focused enrichment-based investigations (Kleifeld et al., 2010), and for phosphorylation the coverage is high enough to use system level analysis tools. For example, the enzymes that catalyze the addition or removal of PTMs often achieve their cellular selectivity by having specificity toward linear sequence motifs in substrate proteins (Puntervoll et al., 2003). To identify enriched sequence motifs among the individual PTM sites identified we used the iceLogo software tool (Colaert et al., 2009). This analysis revealed strong overrepresentation of proline-directed phosphorylation of serine/threonine sites and N-terminal protein acetylation of methionine, alanine, and serine residues (Figure 5A). These observations are in line with previous large-scale proteomics studies of these PTMs reflecting well-known biology, for example the generally high cellular activity and abundance of proline-directed kinases, such as CDKs and MAPKs (Huttlin et al., 2010; Lundby et al., 2012).

Since phosphorylation was the most abundant PTM identified, we determined the completeness of the HeLa phosphoproteome that we achieved without specific enrichment. For comparison, we performed HpH fractionation of a tryptic HeLa digest and enriched phosphopeptides from concatenated fractions using TiO_2 (Batth et al., 2014). From three replicates we identified 30,304 unique phosphorylation sites. This phosphoproteome coverage is close to the deepest analysis of the HeLa phosphoproteome to date, where 38,203 sites were identified by multiple treatments and enrichment strategies (Sharma et al., 2014). Merging these datasets results in a fairly low overlap where the combined HeLa phosphoproteome can be said to contain at least 51,291 phosphorylation sites (Figure S5). Importantly, this indicates that even with enrichment, phosphoproteomics is not close to completeness yet.

Despite the incomplete phosphoproteome coverage without the use of specific phosphopeptide enrichment, quantitative relationships can be derived directly from these data. For example, we calculated the fractional stoichiometry of the individual identified phosphorylation sites by comparing them with their corresponding non-phosphorylated counterpart peptides (Olsen et al., 2010; Sharma et al., 2014). Given the general high sequence coverage we could estimate the stoichiometry for the majority of the phosphorylation sites identified without specific enrichment. The fractional stoichiometry of the sites we identify without specific enrichment were found to be inversely proportional to the abundance of the corresponding protein (Figure 5B). This verifies that we have an abundance bias as sites of low stoichiometry can only be detected on proteins of high abundance and vice versa. Despite this bias, interesting interpretations could be made, and these allowed us to determine if protein kinases have any preferences with regard to the cellular abundance of their substrates. Unsupervised clustering of the HeLa phosphorylation site stoichiometries and their corresponding protein iBAQ values resulted in four main clusters (Figure 5C). Kinase motif analysis of each of the four clusters revealed significant overrepresentation of casein kinase 2 (CK2) substrates in the cluster representing high abundance proteins with high site stoichiometry. This reflects the well-known biology of CK2 that is a ubiquitous, highly pleiotropic, and constitutively active enzyme (Meggio and Pinna, 2003), and therefore phosphorylates

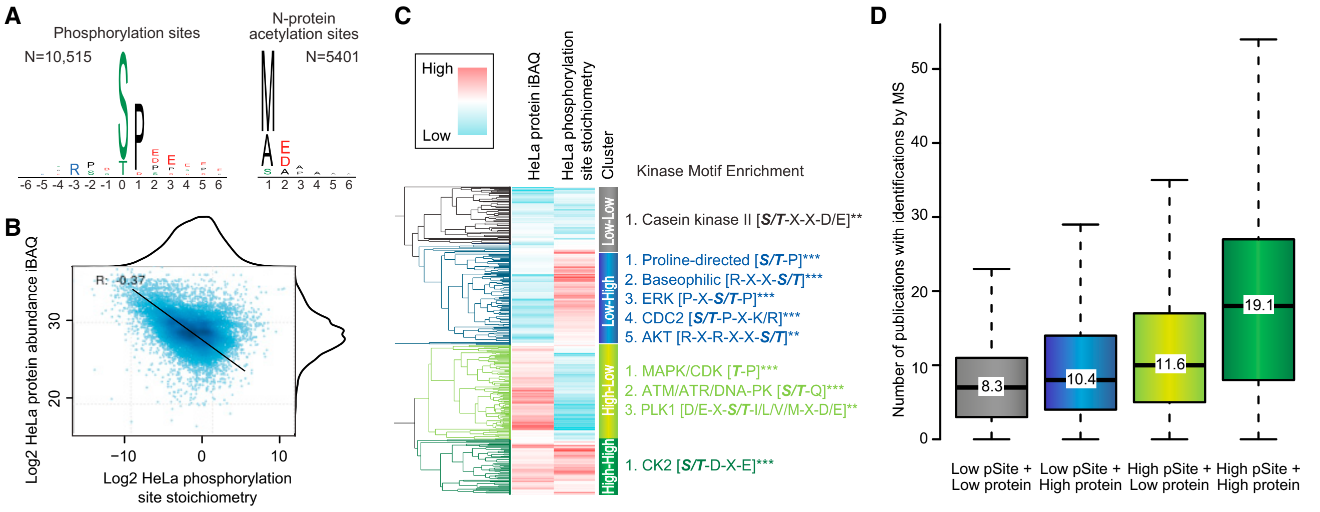

Figure 5. Post-Translational Modifications, PTMs, Identified in HeLa

(A) Sequence logo plots of major PTMs identified in HeLa without specific enrichment.

(B) Correlation between protein abundance and phosphorylation site stoichiometry.

(C) Kinase motif enrichment analysis of four sub-clusters found by comparison of phosphorylation site stoichiometry and their corresponding protein abundance.

(D) Boxplot analysis of citations associated with phosphorylation sites in the four sub-clusters.

its substrates to full site stoichiometry. Conversely, kinase motif analysis of the cluster presenting proteins of high abundance with low phosphorylation site stoichiometry indicated overrepresentation of targets of cell-cycle kinases and DNA damage-response kinases, which are in line with observations from previous studies (Olsen et al., 2010). Notably, the cluster analysis also suggests that proline-directed kinases and baseophilic kinases generally phosphorylate sites on proteins of low abundance to high stoichiometry.

Since its introduction a decade ago global phosphoproteomics has revolutionized the cell signaling field and has become a standard technology accessible in many laboratories producing a wealth of publications (von Stechow et al., 2015). A site-specific citation index can therefore be calculated indicating how frequently a phosphorylation site is reported in the literature (Hornbeck et al., 2004). When this analysis is applied to our clusters of sites, a strong dependence between the site citation index and the abundance of the site and its corresponding protein is revealed (Figure 5D). In other words, low stoichiometry phosphorylation sites in HeLa cells are less studied in the general literature compared with sites we find to be of high abundance. As control, we performed a similar analysis on the proteome as protein citation counts are available (Szklarczyk et al., 2015). This analysis failed to find any dependence between the protein abundance in HeLa cells with how studied the protein is. Collectively, these analyses suggest that future technical challenges specific to phosphoproteomics may arise from this strong abundance bias we find in phosphoproteomics investigations reported so far.

Comprehensive Proteome Analysis of Human Cell Lines and Patient Samples

To assess the general applicability and quantitative accuracy of this trypsin-based multi-shot proteomics workflow, we applied it to five additional cancer cell lines and patient biopsy samples from three different organs (Figure 6A). From all cell lines and tissue samples we collectively identify 724,780 unique peptides, 15,984 protein groups, and 13,446 protein-coding genes (Tables S2, S6, and S7). This deep and comprehensive coverage of peptides and proteins for other cell lines and tissues is similar to the numbers achieved for a comparable single HeLa analysis (Tables S7 and S8). Hierarchical clustering of the label-free quantitation values for all detected proteins across six different cell lines revealed high reproducibility between biological replicates with Pearson correlation coefficients above 0.95 (Figure 6B). We observe that the HEK293 cell proteome expression profile was most similar to that of SY5Y neuroblastoma cells, while the other four cell lines form a separate group. A neuronal expression phenotype of HEK293 cells has been reported previously, which could potentially explain this observation (Shaw et al., 2002). The protein expression profiles for these cancer cell lines are overall very comparable, but important differences are present. Analyzing the expression differences of proteins that are members of specific cellular signaling pathways, such as the cell-cycle network, reveals quantitative differences across the cell lines (Figure 6C). Large abundance differences are observed for key proteins, such as p53, CDN1A, and several protein kinases, indicating a total rewiring of cell division control systems in the individual cell lines. Some of these differences may be known (for example, the high expression level of p53 in HEK293 compared with the other cell lines), but such deep proteome coverage can be used to quantify globally how perturbations of a cellular signaling network may impact protein expression in cancer cell lines.

Notably, this proteomics workflow works equally well to human tissues yielding a similar coverage of peptides and proteins (Table S8). Overlapping our tissue proteomes of liver, colon, and prostate with previously published datasets demonstrates that

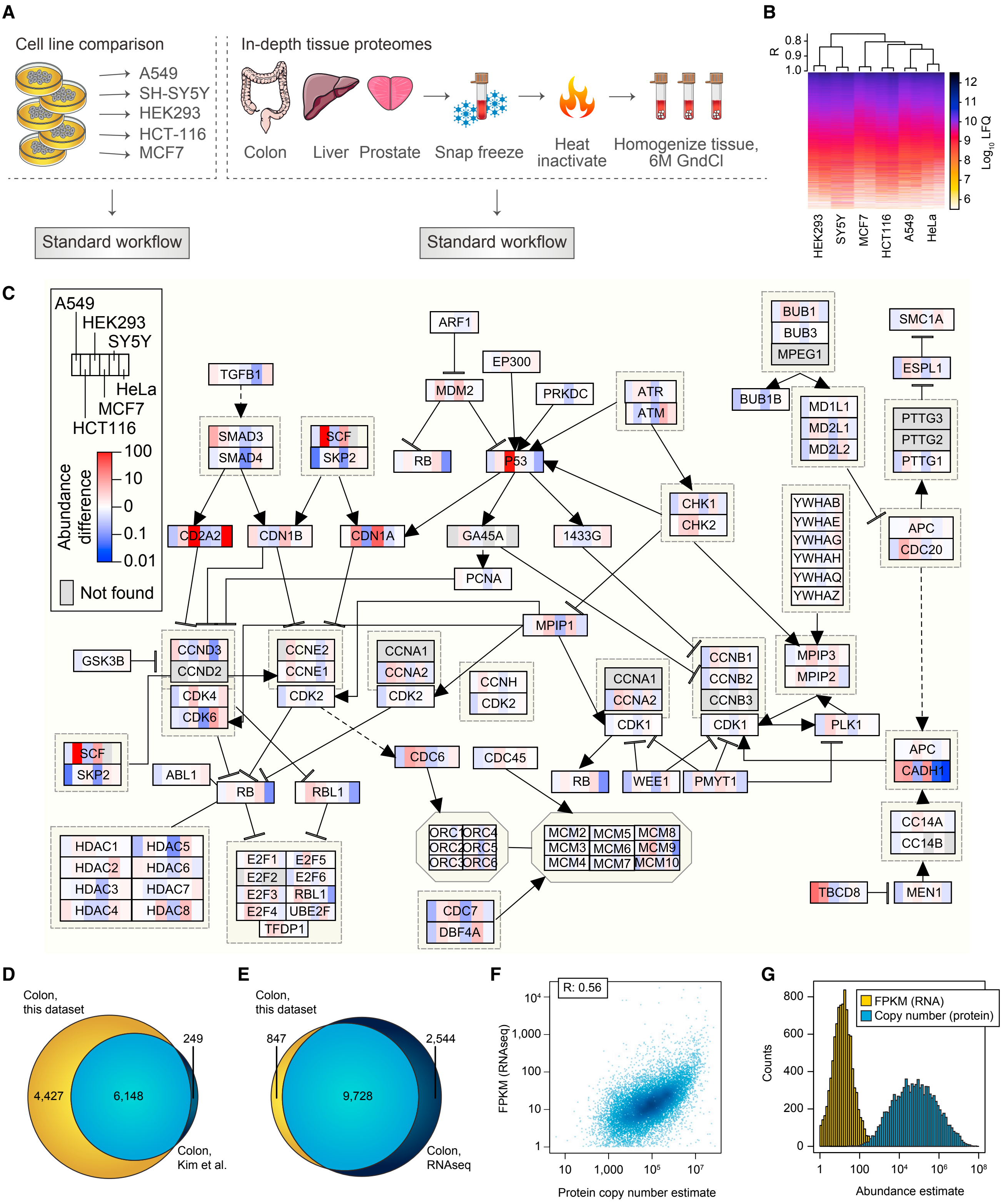

Figure 6. Deep Proteome Analysis of Human Cell Lines and Patient Biopsies

(A) Application of the standard 46 HpH fractions-based workflow to five additional human cell lines and three human tissues.

(B) Hierarchical clustering and heatmap visualization of protein abundances of two replicates for each cell line.

(C) Cell-cycle pathway map with proteins colored according to their relative expression between cell lines.

(legend continued on next page)

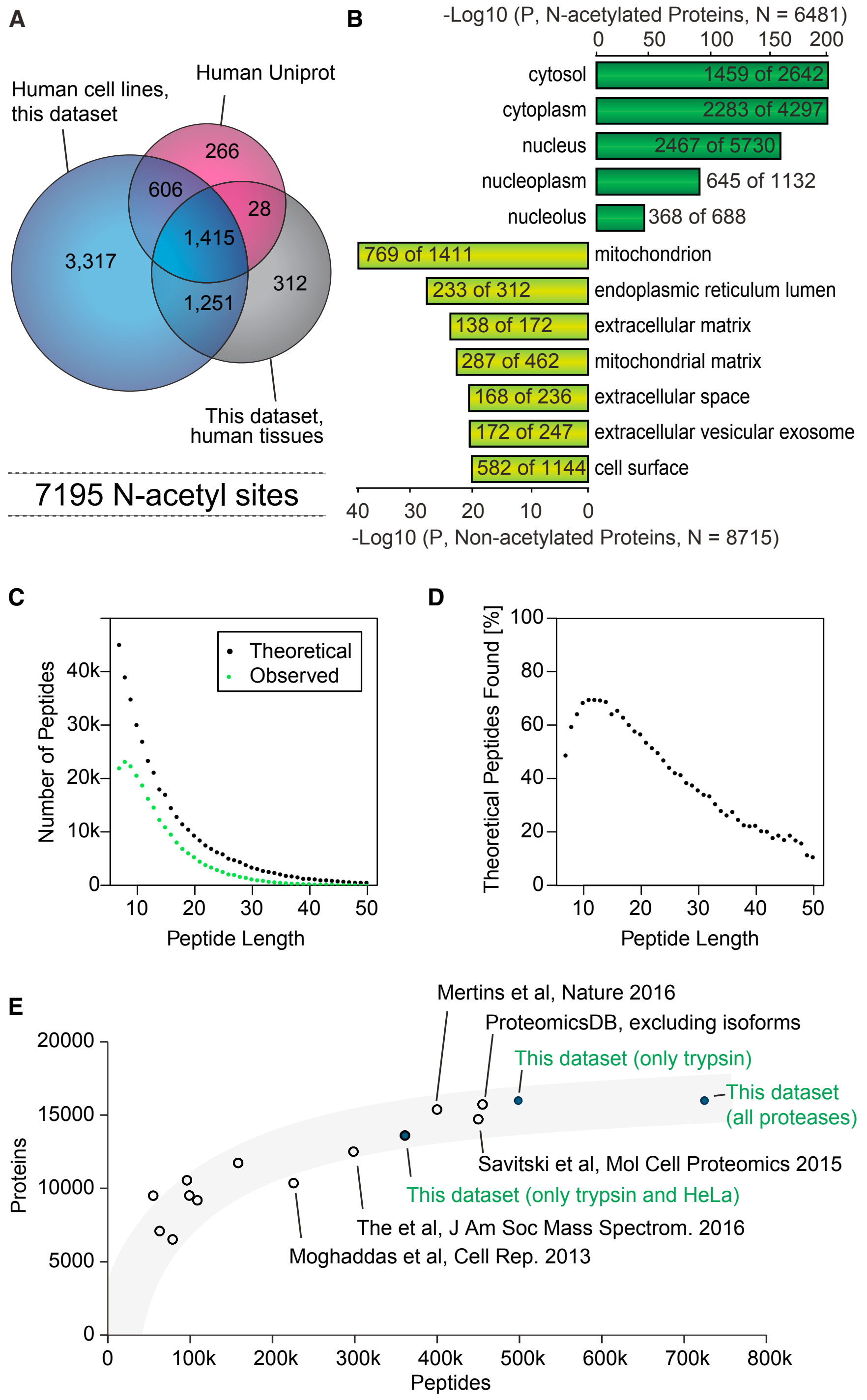

Figure 7. Large-Scale Analysis

(A) Venn diagram showing overlap between proteins with N-acetylation identified in this dataset and annotations in UniProt.

(B) Cellular compartment gene ontology enrichment analysis of the N-acetylated proteome compared with the non-acetylated proteome.

(C) Comparison of observed versus theoretical tryptic peptides from the 12,209 protein-coding genes found in HeLa as a function of peptide length.

(D) Fractional coverage of the theoretical peptide space in HeLa.

(E) Comparison of this dataset with published large-scale proteome datasets with more than 6,500 proteins.

proteome depth of all tissues is increased by thousands of proteins, for example increasing the human colon tissue proteome by an additional 4,400 proteins to 10,500 protein-coding genes (Figure 6D). Next-generation RNA-seq of the same colon patient biopsy combined with a stringent cutoff criteria (fragments per kilobase of transcript per million mapped reads >1) provides evidence for expression of 13,347 different gene products (Figure 6E). This is more genes than we find to be expressed at the protein level, most likely due to only a single replicate being analyzed for each proteome analysis. Despite this limitation, however, the method provides similar depth of the obtained tissue proteomes as those achieved for the cancer cell lines. For a holistic view it is important to analyze both mRNA and protein abundances in parallel as the correlation between gene expression measured by RNA-seq and protein copy numbers estimated by MS was weak, with a Pearson correlation coefficient of 0.52 (Figure 6F). Similar observations have been reported previously (Schwanhausser et al., 2011) in part due to the difference in protein and RNA abundance distribution profiles (Figure 6G).

Collectively our dataset provides a comprehensive resource of high-quality peptide and protein identification and quantitation, which can be mined computationally for new biology. For example, combining all our datasets with the complete human UniProt database reveals an N-acetylated human proteome of more than 7,000 sites of which the majority is covered in the six cell lines analyzed here (Figure 7A). We find N-acetylation sites to be highly enriched on nuclear and cytoplasmic proteins by gene ontology analysis, whereas mitochondrial, extracellular,

(D) Overlap of protein-coding genes identified in colon tissue using this method with a previously published in-depth colon dataset.
(E) Overlap of colon transcriptome and proteome from the same patient sample.
(F) Scatterplot of colon protein copy-number estimates and RNA-seq fragments per kilobase of transcript per million mapped reads (FPKM) values.
(G) Histogram of colon RNA-seq (FPKM) with corresponding proteome copy-number estimates.

and membrane proteins are strongly underrepresented (Figure 7B). Combining all tryptic datasets further underscores the very comprehensive coverage of theoretical tryptic peptides we achieve (Figure 7C). This entire LC-MS workflow is greatly optimized for typical tryptic peptides of length 10–14 as we cover more than 70% of all predicted tryptic peptides in this range (Figure 7D). The combined dataset of only six cell lines and three tissues represents the largest coverage of human proteins and peptides from multi-proteome studies to date (Figure 7E). Importantly, although this workflow has been optimized for starting amounts of 1 mg of peptides, it works equally well for 10-fold lower starting material (Figure S6). This makes it applicable to minute amounts of samples, for example from fluorescence-activated cell-sorted cell populations. The only downside of using less starting material is a significant drop in the number of PTMs, such as phosphorylation sites.

DISCUSSION

The multi-shot proteomics methodological approach presented here represents a straightforward strategy for comprehensive analysis of human proteomes. We circumvent the inherent large dynamic range issues of human proteomes by significantly increasing the peak and loading capacity of our LC-MS system while maintaining overall analysis time. This is achieved by high-resolution offline HpH reversed-phase chromatography with high peptide loads, and collecting a high number of fractions that in turn are analyzed by online low pH LC-MS/MS using fast gradients and fastest peptide sequencing speed. While it has been demonstrated previously that offline HpH reversed-phase fractionation of peptide mixtures in combination with online low pH LC-MS is a powerful technology for deep proteome analysis to a depth of 11,466 protein-coding genes across 20 breast cancer cell lines and 4 tissues (Lawrence et al., 2015), and 12,405 protein-coding genes across 105 tumor samples (Mertins et al., 2016), this study optimizes all parts of the shotgun proteomics workflow to achieve coverage of 12,209 protein-coding genes in a single human cell line covering essentially all expressed proteins and 13,446 protein-coding genes across 6 cell lines and 3 tissues.

An important aspect of deep proteome analysis is control of FDR on protein level as recently debated (Savitski et al., 2015). The MaxQuant software that we used here calculates protein group scores and estimates FDR on both peptide and protein levels, providing an estimate of expected misidentifications at a global level. The publication by Ezkurdia et al. (2014) introduced a simple quality test of large-scale proteome data using the olfactory receptor family. The authors argued that a high-quality proteomics experiment that does not specifically analyze nasal tissues should not expect to detect many peptides for olfactory receptors. Our combined dataset of six cell lines and three tissues presented here, does not contain any olfactory receptors at the 1% protein FDR level, which therefore suggests that it is of a similarly high-quality.

In total, the HeLa proteome presented here represents the first analysis of a human cell line with coverage of protein-coding genes comparable with that of next-generation RNA-seq technologies. Importantly, we demonstrate that our deep comprehensive HeLa proteome is acquired with high quantitative accuracy and that this is independent of the number of offline fractions collected and analyzed. This allowed us to determine the copy number of 14,200 proteins in growing HeLa cells. Given that our dataset represents the largest coverage of tryptic peptides from human proteins to date, it serves as a useful resource of high-resolution MS/MS spectra for the proteomics community. For example, the dataset can be used to generate high-quality and comprehensive spectral libraries for future proteomics experiments. In a recent publication presenting the Human SRMatlas, spectral libraries of 166,174 proteotypic tryptic peptides from all human protein-coding genes were generated by peptide synthesis and MS/MS analyses using quadrupole time-of-flight and triple quadrupole type mass spectrometers (Kusebauch et al., 2016). Here we cover 108,573 of these proteotypic peptides representing two-thirds of the Human SRMatlas, which is the same as our fractional coverage of all human protein-coding genes.

An additional outcome of our deep proteome and improved protein sequence coverage is that we identify thousands of PTMs including more than 10,000 phosphorylation sites. Such deep and broad PTM site coverage normally requires application of specific enrichment strategies in a sequential manner and extensive MS analysis time (Francavilla et al., 2016; Mertins et al., 2013; Swaney et al., 2013), but comes for free with the proteomics workflow presented here. This multi-shot proteomics strategy opens the possibility to study many PTMs simultaneously and estimate their stoichiometry directly (Olsen and Mann, 2013), enabling investigations of PTM crosstalk, an emerging theme in biology (Hunter, 2007), thus providing biological insights into the integrated and complex cell signaling layers.

A current limitation of this approach is that we are still missing comprehensive depth in PTM analysis and quantitative accuracy for proteins of low abundance. But this can likely be addressed in the next generation of MS instrumentation with faster peptide sequencing speed, sensitivity, and increased dynamic range. Nevertheless, we demonstrated the power of this proteomics workflow by applying it to analyze human proteomes from a variety of sample types, including different cancer cell lines and patient organ biopsies. These experiments firmly established the capability of this proteomics strategy delineated here to comprehensively and quantitatively analyze protein expression differences between different cell states. Therefore, the experimental workflow outlined here should be applicable to comprehensively analyze any mammalian cell or tissue proteome in a quantitative manner with sufficient throughput for clinical applications with larger patient cohorts.

STAR★METHODS

Detailed methods are provided in the online version of this paper and include the following:

- KEY RESOURCES TABLE
- CONTACT FOR REAGENT AND RESOURCE SHARING
- EXPERIMENTAL MODEL AND SUBJECT DETAILS
  - Cells
  - Patient Tissue Sample Biopsies
- METHOD DETAILS

- ○ Cell Lysis
- ○ Tissue Homogenization
- ○ Sample Preparation
- ○ Offline High pH Reversed-Phase HPLC Fractionation
- ○ Phosphopeptide Enrichment
- ○ Nanoflow LC–MS/MS
- ○ Next-Generation RNA-Seq
- QUANTIFICATION AND STATISTICAL ANALYSIS
  - ○ Raw Data Processing and Analysis
  - ○ False Discovery Rate (FDR) Analysis
  - ○ Bioinformatics Analysis
- DATA AND SOFTWARE AVAILABILITY

SUPPLEMENTAL INFORMATION

Supplemental Information includes six figures and eight tables and can be found with this article online at http://dx.doi.org/10.1016/j.cels.2017.05.009.

AUTHOR CONTRIBUTIONS

D.B.J. performed the experiments, analyzed data, and contributed to writing the manuscript. T.S.B. established the HpH reversed-phase fractionation system and contributed ideas to data analysis. S.C.L. contributed to samples preparation for experiments described in Figures 6A–6C. K.D.S., C.H., J.B.B., S.H., T.F.Ø., J.B.B., and C.L.A. collected patient samples and generated the RNA-seq data shown in Figures 6D and 6E. M.L.N. edited the manuscript and discussed the results. C.D.K. and J.V.O. designed the experiments, critically evaluated the results, analyzed the data, and wrote the manuscript. All authors read and approved the final version of the manuscript.

ACKNOWLEDGMENTS

The authors thank all lab members for fruitful discussion and input on the manuscript. We thank Associate Prof. Alicia Lundby for help with tissue sample preparation. Work at The Novo Nordisk Foundation Center for Protein Research (CPR) is funded in part by a generous donation from the Novo Nordisk Foundation (grant no. NNF14CC0001). The proteomics technology developments applied was part of a project that has received funding from the European Union's Horizon 2020 Research and innovation program under grant agreement no. 686547. We would like to thank the PRO-MS Danish National Mass Spectrometry Platform for Functional Proteomics and the CPR Mass Spectrometry Platform for instrument support and assistance. J.V.O. was supported by the Danish Cancer Society (R90-A5844 KBVU project grant). The collection and RNA-seq analysis of the human tissues were funded in part through grants from Novo Nordisk Foundation (grant no. NNF14OC0012747), the Danish Cancer Society, and the Danish Council for Independent Research | Medical Science.

Received: January 20, 2017
Revised: March 3, 2017
Accepted: May 11, 2017
Published: June 7, 2017

REFERENCES

Arabi, A., Ullah, K., Branca, R.M.M., Johansson, J., Bandarra, D., Haneklaus, M., Fu, J., Aries, I., Nilsson, P., Den Boer, M.L., et al. (2012). Proteomic screen reveals Fbw7 as a modulator of the NF-kappa B pathway. Nat. Commun. *3*, 976.

Batth, T.S., Francavilla, C., and Olsen, J.V. (2014). Off-line high-pH reversed-phase fractionation for in-depth phosphoproteomics. J. Proteome Res. *13*, 6176–6186.

Beck, M., Schmidt, A., Malmstroem, J., Claassen, M., Ori, A., Szymborska, A., Herzog, F., Rinner, O., Ellenberg, J., and Aebersold, R. (2011). The quantitative proteome of a human cell line. Mol. Syst. Biol. *7*, 549.

Boisvert, F.M., Ahmad, Y., Gierlinski, M., Charriere, F., Lamont, D., Scott, M., Barton, G., and Lamond, A.I. (2012). A quantitative spatial proteomics analysis of proteome turnover in human cells. Mol. Cell. Proteomics *11*, M111.

Branca, R.M.M., Orre, L.M., Johansson, H.J., Granholm, V., Huss, M., Perez-Bercoff, A., Forshed, J., Kall, L., and Lehtio, J. (2014). HiRIEF LC-MSMS enables deep proteome coverage and unbiased proteogenomics. Nat. Methods *11*, 59–62.

Colaert, N., Helsens, K., Martens, L., Vandekerckhove, J., and Gevaert, K. (2009). Improved visualization of protein consensus sequences by iceLogo. Nat. Methods *6*, 786–787.

de Godoy, L.M., Olsen, J.V., Cox, J., Nielsen, M.L., Hubner, N.C., Frohlich, F., Walther, T.C., and Mann, M. (2008). Comprehensive mass-spectrometry-based proteome quantification of haploid versus diploid yeast. Nature *455*, 1251–1254.

Eriksson, J., and Fenyo, D. (2007). Improving the success rate of proteome analysis by modeling protein-abundance distributions and experimental designs. Nat. Biotechnol. *25*, 651–655.

Ezkurdia, I., Vazquez, J., Valencia, A., and Tress, M. (2014). Analyzing the first drafts of the human proteome. J. Proteome Res. *13*, 3854–3855.

Francavilla, C., Papetti, M., Rigbolt, K.T., Pedersen, A.K., Sigurdsson, J.O., Cazzamali, G., Karemore, G., Blagoev, B., and Olsen, J.V. (2016). Multilayered proteomics reveals molecular switches dictating ligand-dependent EGFR trafficking. Nat. Struct. Mol. Biol. *23*, 608–618.

Geiger, T., Wehner, A., Schaab, C., Cox, J., and Mann, M. (2012). Comparative proteomic analysis of eleven common cell lines reveals ubiquitous but varying expression of most proteins. Mol. Cell. Proteomics *11*, M111.

Giansanti, P., Aye, T.T., van den Toorn, H., Peng, M., van Breukelen, B., and Heck, A.J. (2015). An augmented multiple-protease-based human phosphopeptide atlas. Cell Rep. *11*, 1834–1843.

Giansanti, P., Tsiatsiani, L., Low, T.Y., and Heck, A.J. (2016). Six alternative proteases for mass spectrometry-based proteomics beyond trypsin. Nat. Protoc. *11*, 993–1006.

Guillaume, B., Chapiro, J., Stroobant, V., Colau, D., Van Holle, B., Parvizi, G., Bousquet-Dubouch, M.P., Theate, I., Parmentier, N., and Van den Eynde, B.J. (2010). Two abundant proteasome subtypes that uniquely process some antigens presented by HLA class I molecules. Proc. Natl. Acad. Sci. USA *107*, 18599–18604.

Guo, X.F., Trudgian, D.C., Lemoff, A., Yadavalli, S., and Mirzaei, H. (2014). Confetti: a multiprotease map of the HeLa proteome for comprehensive proteomics. Mol. Cell. Proteomics *13*, 1573–1584.

Hanke, S., Besir, H., Oesterhelt, D., and Mann, M. (2008). Absolute SILAC for accurate quantitation of proteins in complex mixtures down to the attomole level. J. Proteome Res. *7*, 1118–1130.

Hebert, A.S., Richards, A.L., Bailey, D.J., Ulbrich, A., Coughlin, E.E., Westphall, M.S., and Coon, J.J. (2014). The one hour yeast proteome. Mol. Cell. Proteomics *13*, 339–347.

Hornbeck, P.V., Chabra, I., Kornhauser, J.M., Skrzypek, E., and Zhang, B. (2004). PhosphoSite: a bioinformatics resource dedicated to physiological protein phosphorylation. Proteomics *4*, 1551–1561.

Hunter, T. (2007). The age of crosstalk: phosphorylation, ubiquitination, and beyond. Mol. Cell *28*, 730–738.

Huttlin, E.L., Jedrychowski, M.P., Elias, J.E., Goswami, T., Rad, R., Beausoleil, S.A., Villen, J., Haas, W., Sowa, M.E., and Gygi, S.P. (2010). A tissue-specific atlas of mouse protein phosphorylation and expression. Cell *143*, 1174–1189.

Iwasaki, M., and Ishihama, Y. (2014). Challenges facing complete human proteome analysis. Chromatography *35*, 73–80.

Iwasaki, M., Sugiyama, N., Tanaka, N., and Ishihama, Y. (2012). Human proteome analysis by using reversed phase monolithic silica capillary columns with enhanced sensitivity. J. Chromatogr. A *1228*, 292–297.

Kelstrup, C.D., Young, C., Lavallee, R., Nielsen, M.L., and Olsen, J.V. (2012). Optimized fast and sensitive acquisition methods for shotgun proteomics on a quadrupole orbitrap mass spectrometer. J. Proteome Res. *11*, 3487–3497.

Kelstrup, C.D., Jersie-Christensen, R.R., Batth, T.S., Arrey, T.N., Kuehn, A., Kellmann, M., and Olsen, J.V. (2014). Rapid and deep proteomes by faster

sequencing on a benchtop quadrupole ultra-high-field Orbitrap mass spectrometer. J. Proteome Res. *13*, 6187–6195.

Kim, D., Pertea, G., Trapnell, C., Pimentel, H., Kelley, R., and Salzberg, S.L. (2013). TopHat2: accurate alignment of transcriptomes in the presence of insertions, deletions and gene fusions. Genome Biol. *14*, R36.

Kim, M.S., Pinto, S.M., Getnet, D., Nirujogi, R.S., Manda, S.S., Chaerkady, R., Madugundu, A.K., Kelkar, D.S., Isserlin, R., Jain, S., et al. (2014). A draft map of the human proteome. Nature *509*, 575–581.

Kleifeld, O., Doucet, A., auf dem Keller, U., and Prudova, A. (2010). Isotopic labeling of terminal amines in complex samples identifies protein N-termini and protease cleavage products. Nature *28*, 281–288.

Kusebauch, U., Campbell, D.S., Deutsch, E.W., Chu, C.S., Spicer, D.A., Brusniak, M.Y., Slagel, J., Sun, Z., Stevens, J., Grimes, B., et al. (2016). Human SRMAtlas: a resource of targeted assays to quantify the complete human proteome. Cell *166*, 766–778.

Kutmon, M., van Iersel, M.P., Bohler, A., Kelder, T., Nunes, N., Pico, A.R., and Evelo, C.T. (2015). PathVisio 3: an extendable pathway analysis toolbox. PLoS Comput. Biol. *11*, e1004085.

Lawrence, R.T., Perez, E.M., Hernández, D., and Miller, C.P. (2015). The proteomic landscape of triple-negative breast cancer. Cell Rep. *11*, 630–644.

Low, T.Y., van Heesch, S., van den Toorn, H., Giansanti, P., Cristobal, A., Toonen, P., Schafer, S., Hubner, N., van Breukelen, B., Mohammed, S., et al. (2013). Quantitative and qualitative proteome characteristics extracted from in-depth integrated genomics and proteomics analysis. Cell Rep. *5*, 1469–1478.

Lundby, A., Secher, A., Lage, K., Nordsborg, N.B., Dmytriyev, A., Lundby, C., and Olsen, J.V. (2012). Quantitative maps of protein phosphorylation sites across 14 different rat organs and tissues. Nat. Commun. *3*, 876.

Masters, J.R. (2002). HeLa cells 50 years on: the good, the bad and the ugly. Nat. Rev. Cancer *2*, 315–319.

Meggio, F., and Pinna, L.A. (2003). One-thousand-and-one substrates of protein kinase CK2? FASEB J. *17*, 349–368.

Mertins, P., Qiao, J.W., Patel, J., Udeshi, N.D., Clauser, K.R., Mani, D.R., Burgess, M.W., Gillette, M.A., Jaffe, J.D., and Carr, S.A. (2013). Integrated proteomic analysis of post-translational modifications by serial enrichment. Nat. Methods *10*, 634–637.

Mertins, P., Mani, D.R., Ruggles, K.V., Gillette, M.A., Clauser, K.R., Wang, P., Wang, X., Qiao, J.W., Cao, S., Petralia, F., et al. (2016). Proteogenomics connects somatic mutations to signalling in breast cancer. Nature *534*, 55–62.

Nagaraj, N., Wisniewski, J.R., Geiger, T., Cox, J., Kircher, M., Kelso, J., Paabo, S., and Mann, M. (2011). Deep proteome and transcriptome mapping of a human cancer cell line. Mol. Syst. Biol. *7*, 548.

Nagaraj, N., Kulak, N.A., Cox, J., Neuhauser, N., Mayr, K., Hoerning, O., Vorm, O., and Mann, M. (2012). System-wide perturbation analysis with nearly complete coverage of the yeast proteome by single-shot ultra HPLC runs on a bench top Orbitrap. Mol. Cell. Proteomics *11*, M111.013722.

Olsen, J.V., and Mann, M. (2013). Status of large-scale analysis of post-translational modifications by mass spectrometry. Mol. Cell. Proteomics *12*, 3444–3452.

Olsen, J.V., Ong, S.E., and Mann, M. (2004). Trypsin cleaves exclusively C-terminal to arginine and lysine residues. Mol. Cell. Proteomics *3*, 608–614.

Olsen, J.V., Macek, B., Lange, O., Makarov, A., Horning, S., and Mann, M. (2007). Higher-energy C-trap dissociation for peptide modification analysis. Nat. Methods *4*, 709–712.

Olsen, J.V., Vermeulen, M., Santamaria, A., Kumar, C., Miller, M.L., Jensen, L.J., Gnad, F., Cox, J., Jensen, T.S., Nigg, E.A., et al. (2010). Quantitative phosphoproteomics reveals widespread full phosphorylation site occupancy during mitosis. Sci. Signal. *3*, ra3.

Ongen, H., Andersen, C.L., Bramsen, J.B., Oster, B., Rasmussen, M.H., Ferreira, P.G., Sandoval, J., Vidal, E., Whiffin, N., Planchon, A., et al. (2014). Putative *cis*-regulatory drivers in colorectal cancer. Nature *512*, 87–90.

Puntervoll, P., Linding, R., Gemund, C., Chabanis-Davidson, S., Mattingsdal, M., Cameron, S., Martin, D.M., Ausiello, G., Brannetti, B., Costantini, A., et al. (2003). ELM server: a new resource for investigating short functional sites in modular eukaryotic proteins. Nucleic Acids Res. *31*, 3625–3630.

Ruepp, A., Brauner, B., Dunger-Kaltenbach, I., Frishman, G., Montrone, C., Stransky, M., Waegele, B., Schmidt, T., Doudieu, O.N., Stumpflen, V., et al. (2008). CORUM: the comprehensive resource of mammalian protein complexes. Nucleic Acids Res. *36*, D646–D650.

Ruepp, A., Waegele, B., Lechner, M., Brauner, B., Dunger-Kaltenbach, I., Fobo, G., Frishman, G., Montrone, C., and Mewes, H.W. (2010). CORUM: the comprehensive resource of mammalian protein complexes – 2009. Nucleic Acids Res. *38*, D497–D501.

Savitski, M.M., Wilhelm, M., Hahne, H., Kuster, B., and Bantscheff, M. (2015). A scalable approach for protein false discovery rate estimation in large proteomic data sets. Mol. Cell. Proteomics *14*, 2394–2404.

Schwanhausser, B., Busse, D., Li, N., Dittmar, G., Schuchhardt, J., Wolf, J., Chen, W., and Selbach, M. (2011). Global quantification of mammalian gene expression control. Nature *473*, 337–342.

Sharma, K., D'Souza, R.C., Tyanova, S., Schaab, C., Wisniewski, J.R., Cox, J., and Mann, M. (2014). Ultradeep human phosphoproteome reveals a distinct regulatory nature of Tyr and Ser/Thr-based signaling. Cell Rep. *8*, 1583–1594.

Shaw, G., Morse, S., Ararat, M., and Graham, F.L. (2002). Preferential transformation of human neuronal cells by human adenoviruses and the origin of HEK 293 cells. FASEB J. *16*, 869–871.

Shishkova, E., Hebert, A.S., and Coon, J.J. (2016). Now, more than ever, proteomics needs better chromatography. Cell Syst. *3*, 321–324.

Spicer, V., Ezzati, P., Neustaeter, H., Beavis, R.C., Wilkins, J.A., and Krokhin, O.V. (2016). 3D HPLC-MS with reversed-phase separation functionality in all three dimensions for large-scale bottom-up proteomics and peptide retention data collection. Anal. Chem. *88*, 2847–2855.

Swaney, D.L., Wenger, C.D., and Coon, J.J. (2010). Value of using multiple proteases for large-scale mass spectrometry-based proteomics. J. Proteome Res. *9*, 1323–1329.

Swaney, D.L., Beltrao, P., Starita, L., Guo, A., Rush, J., Fields, S., Krogan, N.J., and Villen, J. (2013). Global analysis of phosphorylation and ubiquitylation cross-talk in protein degradation. Nat. Methods *10*, 676–682.

Szklarczyk, D., Franceschini, A., Wyder, S., Forslund, K., Heller, D., Huerta-Cepas, J., Simonovic, M., Roth, A., Santos, A., Tsafou, K.P., et al. (2015). STRING v10: protein-protein interaction networks, integrated over the tree of life. Nucleic Acids Res. *43*, D447–D452.

Thakur, S.S., Geiger, T., Chatterjee, B., Bandilla, P., Frohlich, F., Cox, J., and Mann, M. (2011). Deep and highly sensitive proteome coverage by LC-MS/MS without prefractionation. Mol. Cell. Proteomics *10*, M110.

The, M., MacCoss, M.J., Noble, W.S., and Kall, L. (2016). Fast and accurate protein false discovery rates on large-scale proteomics data sets with percolator 3.0. J. Am. Soc. Mass Spectrom. *27*, 1719–1727.

Ting, L., Rad, R., Gygi, S.P., and Haas, W. (2011). MS3 eliminates ratio distortion in isobaric multiplexed quantitative proteomics. Nat. Methods *8*, 937–940.

Trapnell, C., Williams, B.A., Pertea, G., Mortazavi, A., Kwan, G., van Baren, M.J., Salzberg, S.L., Wold, B.J., and Pachter, L. (2010). Transcript assembly and quantification by RNA-seq reveals unannotated transcripts and isoform switching during cell differentiation. Nat. Biotechnol. *28*, 511–515.

Tyanova, S., Temu, T., and Cox, J. (2016a). The MaxQuant computational platform for mass spectrometry-based shotgun proteomics. Nat. Protoc. *11*, 2301–2319.

Tyanova, S., Temu, T., Sinitcyn, P., Carlson, A., Hein, M.Y., Geiger, T., Mann, M., and Cox, J. (2016b). The Perseus computational platform for comprehensive analysis of (prote)omics data. Nat. Methods *13*, 731–740.

Van Hoof, D., Munoz, J., Braam, S.R., Pinkse, M.W.H., Linding, R., Heck, A.J.R., Mummery, C.L., and Krijgsveld, J. (2009). Phosphorylation dynamics during early differentiation of human embryonic stem cells. Cell Stem Cell *5*, 214–226.

Vizcaino, J.A., Deutsch, E.W., Wang, R., Csordas, A., Reisinger, F., Rios, D., Dianes, J.A., Sun, Z., Farrah, T., Bandeira, N., et al. (2014). ProteomeXchange provides globally coordinated proteomics data submission and dissemination. Nat. Biotechnol. *32*, 223–226.

von Stechow, L., Francavilla, C., and Olsen, J.V. (2015). Recent findings and technological advances in phosphoproteomics for cells and tissues. Expert Rev. Proteomics *12*, 469–487.

Wang, Y., Yang, F., Gritsenko, M.A., Clauss, T., Liu, T., Shen, Y., Monroe, M.E., Lopez-Ferrer, D., Reno, T., Moore, R.J., et al. (2011). Reversed-phase chromatography with multiple fraction concatenation strategy for proteome profiling of human MCF10A cells. Proteomics *11*, 2019–2026.

Wilhelm, M., Schlegl, J., Hahne, H., Moghaddas Gholami, A., Lieberenz, M., Savitski, M.M., Ziegler, E., Butzmann, L., Gessulat, S., Marx, H., et al. (2014). Mass-spectrometry-based draft of the human proteome. Nature *509*, 582–587.

Wisniewski, J.R., Zougman, A., Nagaraj, N., and Mann, M. (2009). Universal sample preparation method for proteome analysis. Nat. Methods *6*, 359–362.

Wisniewski, J.R., Hein, M.Y., Cox, J., and Mann, M. (2014). A "proteomic ruler" for protein copy number and concentration estimation without spike-in standards. Mol. Cell. Proteomics *13*, 3497–3506.

Wolters, D.A., Washburn, M.P., and Yates, J.R., 3rd (2001). An automated multidimensional protein identification technology for shotgun proteomics. Anal. Chem. *73*, 5683–5690.

Yamana, R., Iwasaki, M., Wakabayashi, M., Nakagawa, M., Yamanaka, S., and Ishihama, Y. (2013). Rapid and deep profiling of human induced pluripotent stem cell proteome by one-shot Nanolc-MS/MS analysis with meter-scale monolithic silica columns. J. Proteome Res. *12*, 214–221.

Zeiler, M., Straube, W.L., Lundberg, E., Uhlen, M., and Mann, M. (2012). A Protein Epitope Signature Tag (PrEST) library allows SILAC-based absolute quantification and multiplexed determination of protein copy numbers in cell lines. Mol. Cell. Proteomics *11*, O111.009613.

STAR★METHODS

KEY RESOURCES TABLE

REAGENT or RESOURCE	SOURCE	IDENTIFIER
Biological Samples		
Prostate patient biopsy	Århus University Hospital	N/A
Colon patient biopsy	Århus University Hospital	N/A
Liver patient biopsy	Århus University Hospital	N/A
Chemicals, Peptides, and Recombinant Proteins		
DMEM	Gibco	31966-047
RPMI	Gibco	61870-010
Fetal Bovine Serum (heat-inactivated)	Gibco	10270-106
Pen/strep	Gibco	15140-122
Trypsin-EDTA (0.05%)	Gibco	25300-054
PBS	Gibco	20012-068
Guanidine hydrochloride	Sigma Aldrich	G3272
TCEP	Sigma Aldrich	C4706
CAA	Sigma Aldrich	22790
Trizma base	Sigma Aldrich	T1503
RNeasy Mini Kit	Qiagen	74104
Complete mini EDTA-free protease inhibitor cocktail	Roche	04693124001
Trypsin	Sigma Aldrich	T6567
Lys-C	Wako Chemicals	129-02541
Glu-C	Roche	11047817001
Chymotrypsin	Roche	11418467001
TFA	Sigma Aldrich	T6508
Acetonitrile	Merck	1.00030.2500
Ammonium bicarbonate	Sigma Aldrich	09830
5μM Titansphere	GL Sciences	GS 502075000
2,5-dihydroxybenzoic acid	Sigma Aldrich	85707
Ammonia solution 25%	Merck	1054321011
Deposited Data		
Raw and analyzed data	This paper	ProteomeXchange: PXD004452 http://proteomecentral.proteomexchange.org/cgi/GetDataset?ID=PXD004452
Experimental Models: Cell Lines		
HeLa	ATCC	CCL-2
A549	ATCC	CCL-185
MCF7	ATCC	HTB-22
SH-SY5Y	Sven Lindner (Germany)	N/A
HEK293T	Jiri Lukas (Copenhagen)	N/A
HCT116	ATCC	CCL-247
Software and Algorithms		
MaxQuant 1.5.3.6	N/A	http://www.coxdocs.org/doku.php?id=maxquant:start
R software	N/A	https://www.r-project.org/
Perl	N/A	https://www.perl.org/
PathVisio 3.2.2	N/A	https://www.pathvisio.org/

(*Continued on next page*)

Continued

REAGENT or RESOURCE	SOURCE	IDENTIFIER
String v10	N/A	http://string-db.org/
Cytoscape	N/A	www.cytoscape.org
IceLogo	N/A	http://iomics.ugent.be/icelogoserver/index.html

CONTACT FOR REAGENT AND RESOURCE SHARING

Further information and requests for resources and reagents should be directed to the Lead Contact, Jesper V. Olsen, by email at jesper.olsen@cpr.ku.dk

EXPERIMENTAL MODEL AND SUBJECT DETAILS

Cells

Human epithelial cervix carcinoma HeLa cells (female), human embryonic kidney HEK293 cells (fetus), human neuroblastoma SH-SY5Y cells (female), lung adenocarcinoma A549 cells (male), human colon cancer HTC116 cells and human breast cancer MCF-7 cells (female) were purchased from ATCC. Cells were cultured in DMEM (Gibco, Invitrogen), supplemented with 10% fetal bovine serum, 100U/mL penicillin (Invitrogen), 100μg/mL streptomycin (Invitrogen), at 37°C, in a humidified incubator with 5% $CO2$. SH-SY5Y was cultured in RPMI (Gibco, Invitrogen) with the same supplements as listed above. We have not performed specific authentication of the cell lines used in this study.

Patient Tissue Sample Biopsies

Collection and use of the human samples provided by Department of Molecular Medicine, Aarhus University Hospital, Aarhus, Denmark has been approved by The Central Denmark Region Committees on Health Research Ethics (J. no"s M-1999/4678 and M-2000/0299). Informed consent was obtained from all donors. Use of clinical data associated with the samples has been approved by the Danish Data protection Agency (j. no 2007-58-0010 and j.no. 2013-41-2041). Total RNA from serial cryosections were extracted using the RNeasy Mini Kit (Qiagen). RNA integrity was assessed using the Agilent RNA 6000 Nano Kit on an Agilent 2100 Bioanalyzer and the analyzed samples had RNA integrity numbers (RIN) >9.

METHOD DETAILS

Cell Lysis

Cells were harvested at approximately 80 % confluency by washing twice with PBS (Gibco, Life technologies) and subsequently adding boiling lysis buffer (6 M guanidinium hydrochloride (GndCl), 5 mM tris(2-carboxyethyl)phosphine, 10 mM chloroacetamide, 100 mM Tris, pH 8.5) directly to the plate. Cells were collected by scraping the plate and boiled for additional 10 min followed by micro tip sonication.

Tissue Homogenization

Human tissues were quickly dissected and snap frozen. Followed by heat inactivation (Denator T1 Heat Stabilizor, Denator) the tissues were transferred to a GndCl solution (6 M GndCl, 25 mM Tris, pH 8.5, Roche Complete Protease Inhibitor tablets (Roche)) and homogenized by ceramic beads using 2 steps of 20 s at 5500 rpm (Precellys 24, Bertin Technologies). The tissues were heated for 10 min at 95 °C followed by micro tip sonication on ice and clarified by centrifugation (20 min, 16,000g, 4 °C). Samples were reduced and alkylated by adding 5 mM tris(2-carboxyethyl)phosphine and 10 mM chloroacetamide for 20 min at room temperature.

Sample Preparation

Protein concentration was estimated by Bradford assay (Bio-Rad), and the lysates were digested with Lys-C (Wako) in an enzyme/protein ratio of 1:100 (w/w) for 1 h followed by a threefold dilution with 25 mM Tris, pH 8.5, to 2 M GndCl and further digested overnight with trypsin (Sigma Aldrich) 1:100 (w/w). For experiments using different proteases, lysates were directly diluted to 2 M GndCl before addition of proteases (Lys-C, Trypsin, Chymotrypsin (Roche) and Glu-C (Roche)). Protease activity was quenched by acidification with trifluoroacetic acid (TFA) to a final concentration of approximately 1%, and the resulting peptide mixture was concentrated using reversed-phase Sep-Pak C18 Cartridge (Waters). Peptides were eluted off the Sep-Pak with 2 mL 40% acetonitrile (ACN) followed by 2 mL 60% ACN. The ACN was removed by vacuum centrifugation for 40 min at 60 °C and the final peptide concentration was estimated by measuring absorbance at 280 nm on a NanoDrop (NanoDrop 2000C, Thermo Scientific).

Offline High pH Reversed-Phase HPLC Fractionation

1-2 mg of peptides were fractionated using a Waters XBridge BEH130 C18 3.5 μm 4.6 × 250 mm column on an Ultimate 3000 high-pressure liquid chromatography (HPLC) system (Dionex, Sunnyvale, CA, USA) operating at a flow rate of 1 mL/min with three buffer lines: Buffer A consisting of water, buffer B of ACN and Buffer C of 25 mM Ammonium bicarbonate, pH8. Peptides were separated by a linear gradient from 5% B to 35% B in 62 min followed by a linear increase to 60% B in 5 min, and ramped to 70% B in 3 min. Buffer C was constantly introduced throughout the gradient at 10%. Fractions were collected at 60 s or 90 s intervals to a total of either 39, 46, or 70 fractions. Samples were acidified with formic acid to a final concentration of approximately 0.1% prior to concentration using vacuum centrifugation. For nanoflow LC–MS/MS, the loading amount was kept constant at 1 μg per injection, estimated by measuring absorbance at 280 nm on a NanoDrop instrument.

Phosphopeptide Enrichment

Phosphopeptides from all 46 fractions were enriched using titanium dioxide beads (5 μm Titansphere, GL Sciences, Japan). TiO2 beads were pre-incubated in 2,5-dihydroxybenzoic acid (20 mg/mL) in 80% ACN and 1% TFA (5 μL/mg of beads) for 20 min. All fractions were brought to 80% ACN and 5% TFA in a final volume of 5 mL. 1 mg (in 5μL of DHB solution) was added to each fraction, which was then incubated for 30 min while rotating. After incubation, fractions were transferred to a deep 96-well filter plate and the supernatant was removed by a vacuum manifold and collected to four new fractions with a traditional concatenation scheme. These were incubated with fresh TiO2 beads for a second enrichment step. Beads were washed on the filter plate with 1 mL 80% ACN and 1% TFA, followed by 50% ACN and 1% TFA. The final washing step was with 10% ACN and 1% TFA. The phosphopeptides were eluted from the plate with 400 μL 5% NH_4OH followed by 400 μL 10% NH_4OH with 25% ACN. Eluted peptides were concentrated in a speed-vac and loaded onto C18 Stage-tips.

Nanoflow LC–MS/MS

All samples were analyzed on an Easy-nLC 1000 coupled to a Q-Exactive HF instrument (Thermo Fisher Scientific) equipped with a nanoelectrospray source. Peptides were separated on a 15 cm analytical column (75 μm inner diameter) in-house packed with 1.9 μm C18 beads (Dr. Maisch, Germany). The column temperature was maintained at 40 °C using an integrated column oven (PRSO-V1, Sonation GmbH, Biberach, Germany). Each peptide fraction was auto-sampled and separated using a 30 min gradient ranging from 10 % buffer B (80% ACN and 0.1% formic acid) to 30% B in 25 min and ramped to 45 % B in 5 min at a flow rate of 350 nL/min. The washout followed at 80% buffer B for 4 min. The Q-Exactive HF mass spectrometer was operated in data-dependent acquisition mode. Spray voltage was set to 2 kV, s-lens RF level at 50, and heated capillary temperature at 275 °C. All experiments were performed in the data-dependent acquisition mode to automatically isolate and fragment topN multiply-charged precursors according to their intensities. Former target ions were dynamically for 30 seconds excluded and all experiments were acquired using positive polarity mode. Full scan resolution was set to 60,000 at m/z 200 and the mass range was set to m/z 350-1400. Full scan ion target value was 3E6 allowing a maximum fill time of 100 ms. Higher-energy collisional dissociation (HCD)(Olsen et al., 2007) fragment scans was acquired with optimal setting for parallel acquisition using 1.3 m/z isolation width and normalized collision energy of 28. For fastest HCD-MS/MS scanning a top20 method was employed with fragment scan resolution of 15,000 and an ion target value of 1E5 allowing maximum filling time of 15 ms. For protease comparisons, LC-MS/MS experiments were analyzed with a mix of 30 min and 60 min LC gradients and full scan resolutions at 120,000 at m/z 200 with a maximum fill time of 25 ms. For 60 min gradients, fast scanning top12 method using 30,000 resolution for HCD scans with maximum fill time of 45 ms was acquired. Phosphopeptide-enriched samples were analyzed with a sensitive top7 scanning method. Ion target value for HCD fragment scans were set to 2E5 with a maximum fill time of 110 ms and analyzed with 60,000 resolution.

Next-Generation RNA-Seq

Paired end mRNA sequencing was performed as previously described (Ongen et al., 2014) using the Illumina Hiseq 2000 Platform. In brief, 500 ng total RNA was used for library preparation with the TruSeq RNA Sample Prep Kit v2 and the libraries had fragment lengths of ~200bp. TruSeq PE Cluster Kit v3 was used for cluster generation, and TruSeq SBS Kit v3 for sequencing. Human transcriptome quantification was performed by trimming read adaptor sequences using the AdapterRemoval tool (https://github.com/slindgreen/AdapterRemoval) mapping the reads to the human genome issue HG19 (hg19) using the Tophat2 mapper (Kim et al., 2013) and estimating FPKM values for Ensembl genes using Cufflink (Gencode v15 annotation w/o Pseudogenes) (Trapnell et al., 2010).

QUANTIFICATION AND STATISTICAL ANALYSIS

Raw Data Processing and Analysis

All raw LC–MS/MS data were analyzed by MaxQuant v1.5.3.6 using the Andromeda Search engine and searched against the complete human UniProt database including all Swiss-Prot and TrEMBL entries as well as all isoforms. In addition, the default contaminant protein database was included and any hits to this excluded from further analysis. The second peptide option was disabled and “match between runs” features were excluded in the downstream analysis. Two previously published dataset were included as raw-files in our combined MaxQuant analysis. These are three 14 fraction HeLa experiments (Kelstrup et al., 2014) and three human tissues (Kim et al., 2014). Four analysis groups were made in MaxQuant, enabling one combined analysis for all

proteases. Carbamidomethylation of cysteine was specified as fixed modification for all groups. Variable modifications considered were oxidation of methionine, protein N-terminal acetylation, pyro-glutamate formation from glutamine and phosphorylation of serine, threonine, and tyrosine residues. For PTM analysis, the HeLa dataset was searched separately using methylation of argnines and lysines or acetylation of lysines as variable modifications.

False Discovery Rate (FDR) Analysis

The false discovery rate (FDR) was set to 1% on PSM, PTM site and Protein level. The FDR control employed in MaxQuant has recently been described in detail (Tyanova et al., 2016a). Briefly, MaxQuant make use of the target-decoy search strategy to estimate and control the extent of false-positive identifications using the concept of posterior error probability (PEP) to integrate multiple peptide properties, such as length, charge, number of modifications, and Andromeda score into a single quantity reflecting the quality of a peptide spectrum match (PSM). A second level of FDR control is set on the list of reported protein groups by calculating a *Protein group score*. This is the product of individual PEPs of the peptides of a protein group, and includes a factor to take into account the number of peptides per protein group. The protein group score is similar to the PEP, in that it provides a measure of the certainty of protein identification.

When analyzing multiple different cell lines, tissue samples and external datasets together in MaxQuant compared to analyzing the datasets individually, the main difference lies in the calculation of the protein FDR, which is done globally based on all raw files. This is important because if search results for separate raw files or datasets are combined into one larger dataset without any further higher-level FDR control; false-positive protein identifications are known to aggregate. As the computational performance of MaxQuant scales very well with the number of raw files it is recommended to always analyze all raw files that will later be used in a comparative manner together in a single MaxQuant. This has the additional advantage that the protein groups are defined in common for the whole data set, which simplifies quantitative comparative analysis of protein ratios and intensities.

Bioinformatics Analysis

The majority of the bioinformatics was accomplished using custom Perl and R scripts supplemented with Perseus (Tyanova et al., 2016b). Pathway visualization was done using PathVisio 3.2.2 (Kutmon et al., 2015). The String (Szklarczyk et al., 2015) database version 10 was read into Cytoscape (www.cytoscape.org) for visualization of complexes. External datasets used were proteome and RNAseq data from HeLa (Nagaraj et al., 2011), the Corum (Ruepp et al., 2010) database release February 2012 limited to human species, citation numbers were obtained from PhosphoSite.org (Hornbeck et al., 2004), and HeLa copy number estimates (Hanke et al., 2008; Zeiler et al., 2012). Mapping of gene and protein identifiers between experiments were done as follows. All identifiers from UniProt, IPI, and older Ensembl identifiers were mapped to protein-coding genes in the Ensembl database version 84 primary assembly based on GRCh38. The primary gene identifier for each protein group was defined as the ones that represented proteins that could explain all peptides within a protein group. Overlap between datasets was calculated based on match to primary gene identifier. When a gene identifier represents multiple protein groups, the highest number of razor and unique peptides is used to choose the group of the main protein-coding gene. PTM site sequence motif analysis was performed using IceLogo (Colaert et al., 2009)with fold-change as the scoring system and a p-value cut-off of 0.05. Our input dataset was sequence windows for individual PTM sites identified and the complete human dataset were used as the background dataset.

DATA AND SOFTWARE AVAILABILITY

All raw mass spectrometric data files from this study have been deposited to the ProteomeXchange Consortium via the PRIDE partner repository (Vizcaino et al., 2014) with the dataset identifier PXD004452.

Cell Systems, Volume *4*

Supplemental Information

An Optimized Shotgun Strategy

for the Rapid Generation

of Comprehensive Human Proteomes

Dorte B. Bekker-Jensen, Christian D. Kelstrup, Tanveer S. Batth, Sara C. Larsen, Christa Haldrup, Jesper B. Bramsen, Karina D. Sørensen, Søren Høyer, Torben F. Ørntoft, Claus L. Andersen, Michael L. Nielsen, and Jesper V. Olsen

Figure S1

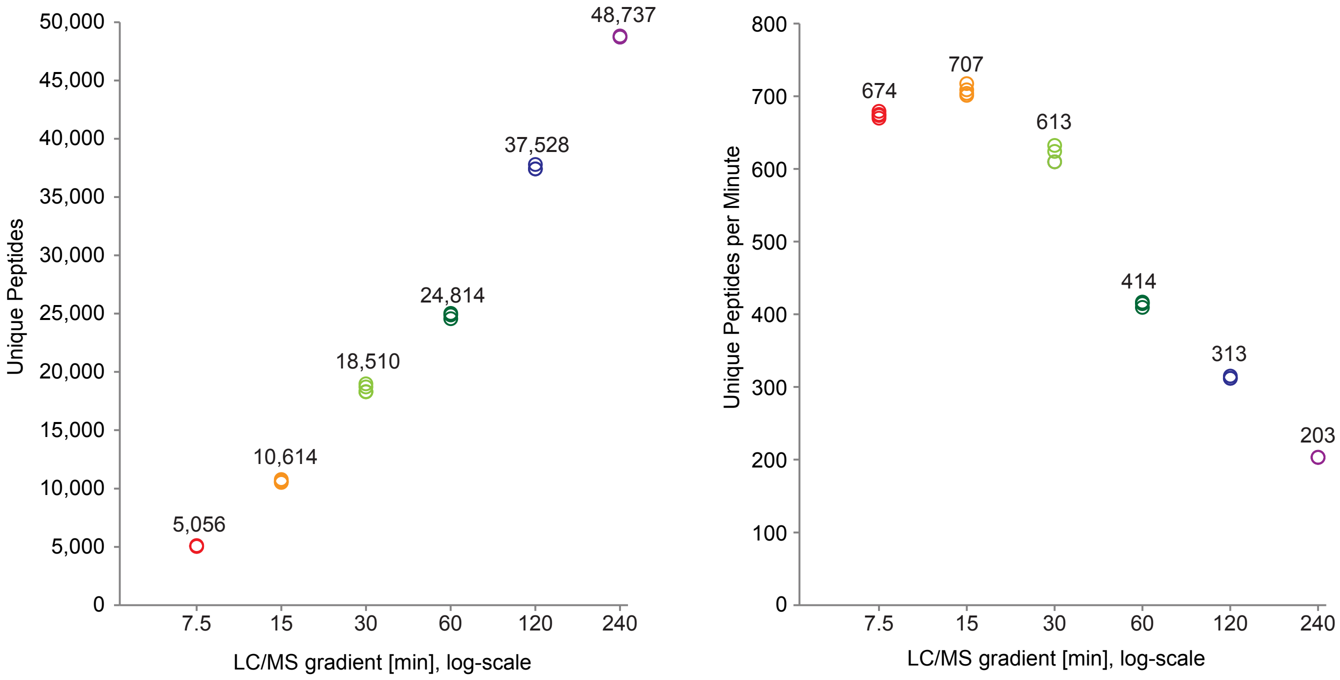

Figure S1: Relationship between LC/MS gradient length and peptide identification rates. Related to Figure 1. A fixed amount of 1ug Hela tryptic digest ‘on column’ was analyzed with variable gradient lengths. Optimal column and MS method was chosen for each time point. Left plot shows the total amount of unique peptides identified. The right plot shows the number of unique peptide identifications per minute. Above each time point the average of replica analysis is shown.

Figure S2

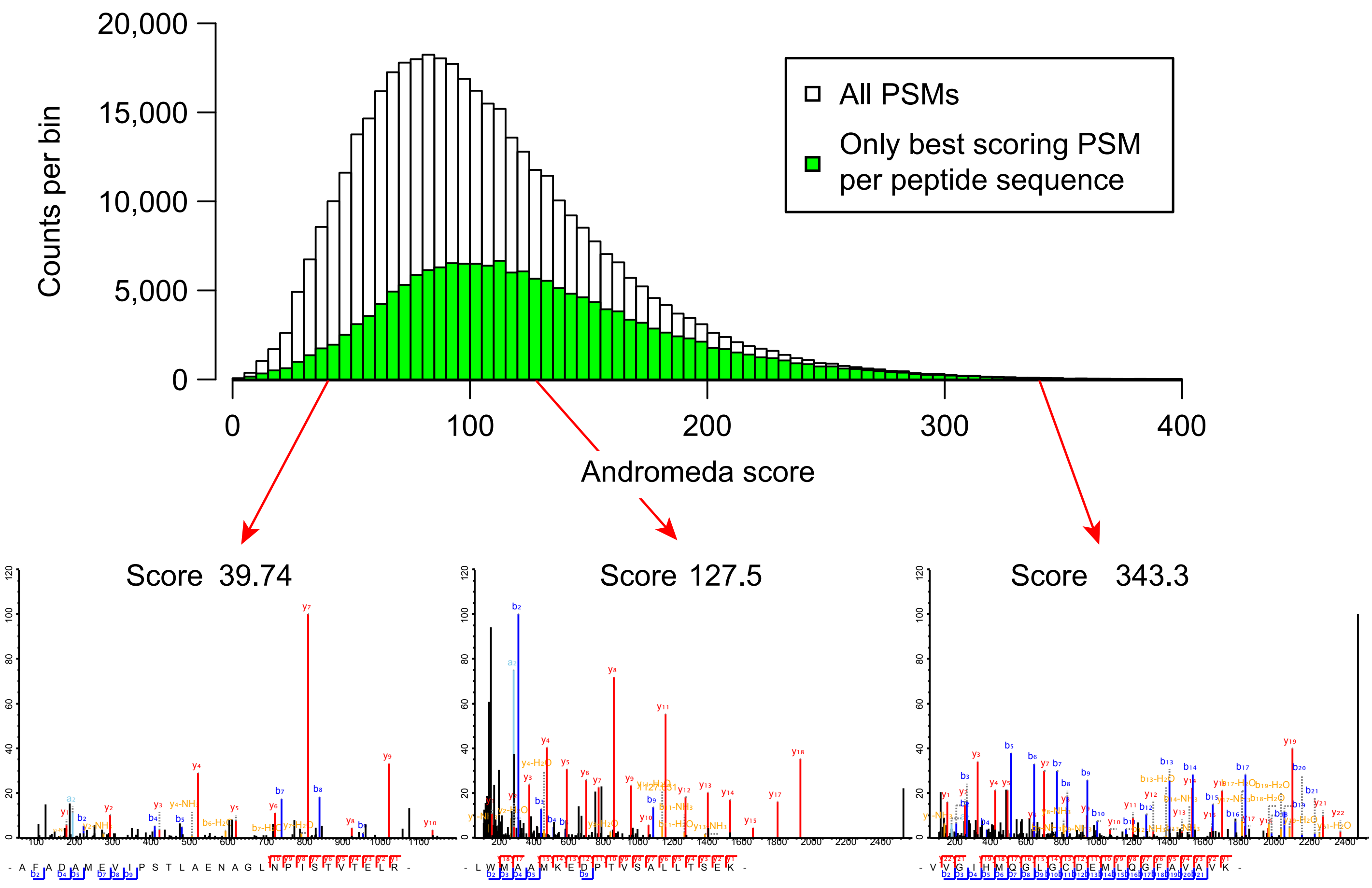

Figure S2: Score distributions for the HeLa experiment fractionated in 46 fractions. Related to Figure 1. The search engine (Andromeda) score distribution is shown for all PSMs and only the best scoring PSM per unique peptide sequence. Further, three annotated example spectra are shown for different scores.

Figure S3

a

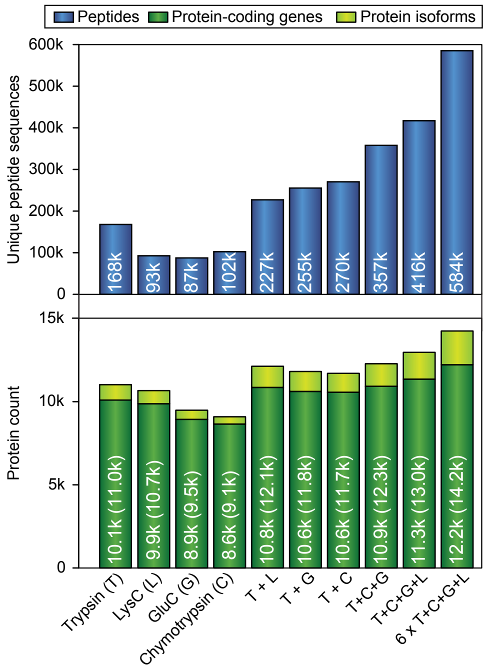

b c

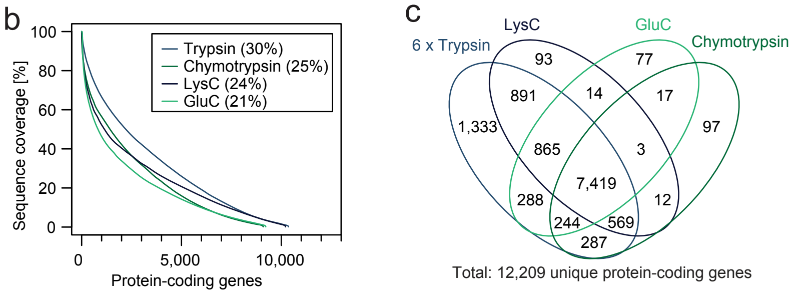

d

Protease	MS/MS	PSMs[a]	ID-rate	Unique Peptides[b]	Theoretical Peptides[c]
Trypsin	1,098,347	426,488	38.9%	168,569	1,866,929
Chymotrypsin	1,265,588	207,681	16.4%	102,311	2,646,893
GluC	1,252,604	249,340	19.9%	87,367	1,384,403
LysC	1,180,428	328,601	27.8%	92,546	1,151,077

[a]Non-redundant PSMs. [b]Unique peptide sequences. [c]Theoretical peptides obtained by in-silico digestion of 12,200 protein coding genes found in HeLa allowing up to 2 missed cleavages and a length above 6 amino acids.

Figure S3: Comparison of proteome coverage with different proteases. Related to Figure 3. a) Peptide and protein comparison using different enzymes. Single and two way combination with trypsin and technical replicates of digests are provided. b) Sequence coverage comparison of the different proteases. c) Venn diagram of protein coding genes between proteases. d) Comparisons of technical details associated with each protease. The identification rate is the proportion of peptide spectrum matches (PSMs) relative to the total number of MS/MS events.

Figure S4

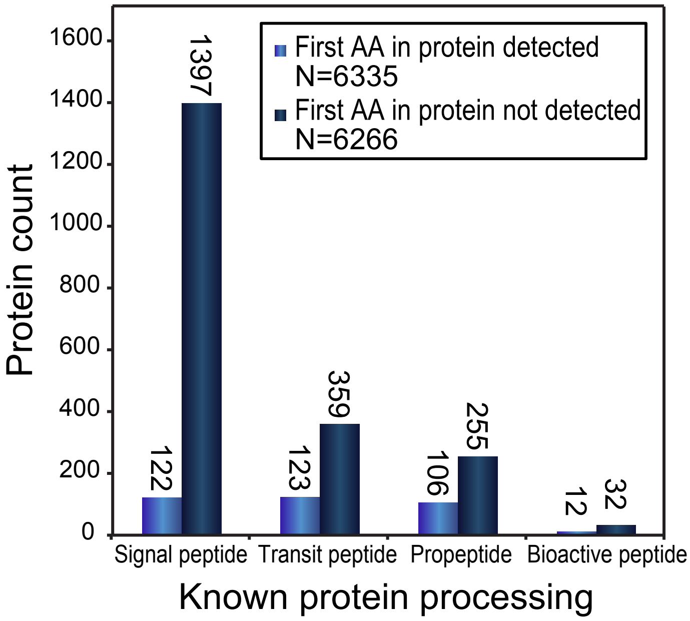

Figure S4: Analysis of protein N-terminus. Related to Figure 5. The two colours indicate whether an identified protein also had peptide sequence coverage of the first amino acid (AA) counted from the N-terminal of the protein. This analysis was extended to include different groups of known protein processing, obtained through annotation of the identified proteins from UniProt.

Figure S5

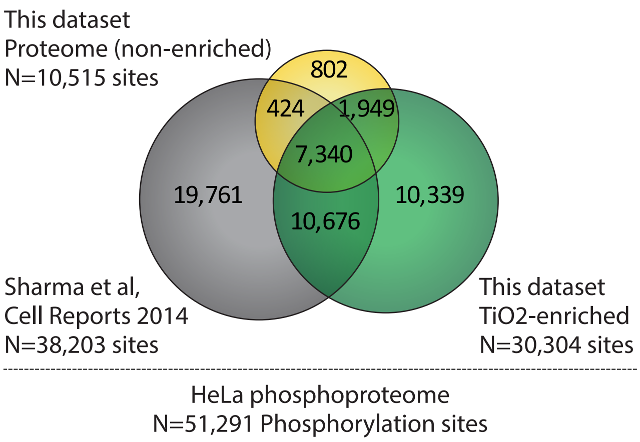

Figure S5: Phosphopeptides in HeLa. Related to Figure 5. Overlap of the phosphorylation sites foun with and without enrichment compared to the largest HeLa phosphoproteome published to date.

Figure S6

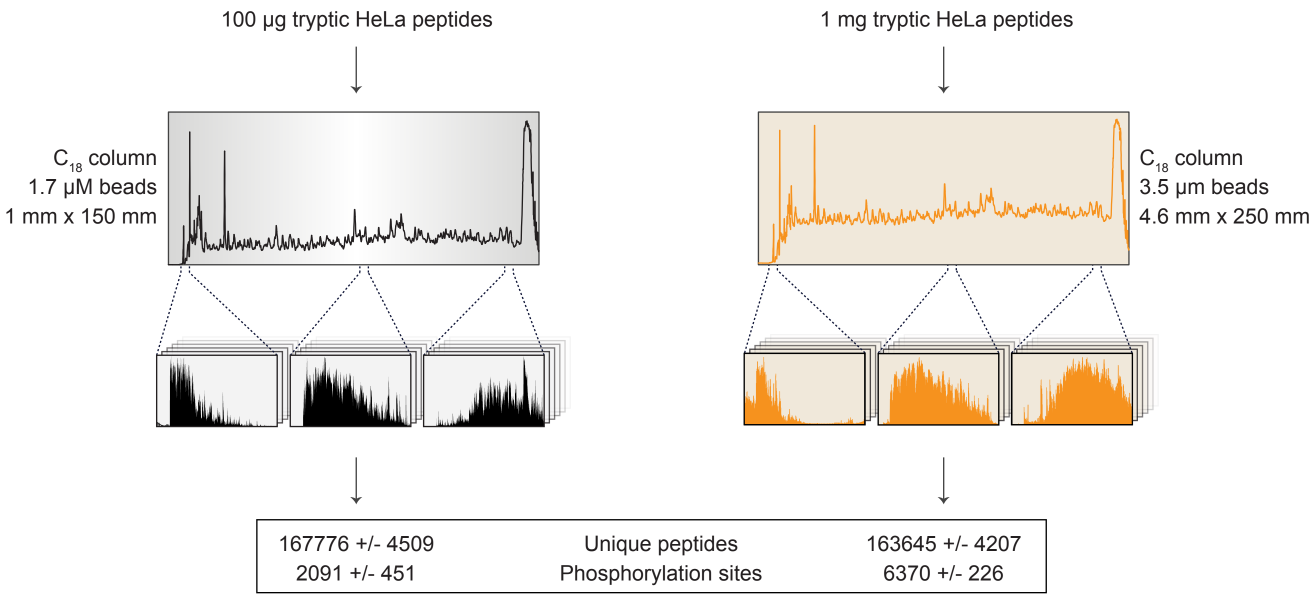

Figure S6: Downscaling of the peptide amount input. Related to Figure 7. Comparison of 100 µg tryptic HeLa peptides with 1 mg using the standard HpH 46 fractionation scheme.
